# Pulmonary surfactant and prostaglandin E_2_ in airway smooth muscle relaxation of human and male guinea pigs

**DOI:** 10.14814/phy2.70026

**Published:** 2024-09-08

**Authors:** J. Hanusrichterova, M. Kolomaznik, R. Barosova, J. Adamcakova, D. Mokra, J. Mokry, H. Skovierova, M. M. Kelly, E. de Heuvel, S. Wiehler, D. Proud, H. Shen, P. G. Mukherjee, M. W. Amrein, A. Calkovska

**Affiliations:** ^1^ Biomedical Centre Martin, Jessenius Faculty of Medicine in Martin Comenius University in Bratislava Martin Slovakia; ^2^ Department of Physiology, Jessenius Faculty of Medicine in Martin Comenius University in Bratislava Martin Slovakia; ^3^ Department of Pharmacology, Jessenius Faculty of Medicine in Martin Comenius University in Bratislava Martin Slovakia; ^4^ Department of Pathology and Laboratory Medicine Cumming School of Medicine, University of Calgary Calgary Alberta Canada; ^5^ Department of Physiology and Pharmacology and Snyder Institute for Chronic Diseases Cumming School of Medicine, University of Calgary Calgary Alberta Canada; ^6^ Department of Mathematics and Statistics, Faculty of Science University of Calgary Calgary Alberta Canada; ^7^ Department of Cell Biology and Anatomy Cumming School of Medicine, University of Calgary Calgary Alberta Canada

**Keywords:** airway smooth muscle, atomic force microscopy, prostaglandin E_2_, pulmonary surfactant, relaxation

## Abstract

Pulmonary surfactant serves as a barrier to respiratory epithelium but can also regulate airway smooth muscle (ASM) tone. Surfactant (SF) relaxes contracted ASM, similar to β_2_‐agonists, anticholinergics, nitric oxide, and prostanoids. The exact mechanism of surfactant relaxation and whether surfactant relaxes hyperresponsive ASM remains unknown. Based on previous research, relaxation requires an intact epithelium and prostanoid synthesis. We sought to examine the mechanisms by which surfactant causes ASM relaxation. Organ bath measurements of isometric tension of ASM of guinea pigs in response to exogenous surfactant revealed that surfactant reduces tension of healthy and hyperresponsive tracheal tissue. The relaxant effect of surfactant was reduced if prostanoid synthesis was inhibited and/or if prostaglandin E_2_‐related EP_2_ receptors were antagonized. Atomic force microscopy revealed that human ASM cells stiffen during contraction and soften during relaxation. Surfactant softened ASM cells, similarly to the known bronchodilator prostaglandin E_2_ (PGE_2_) and the cell softening was abolished when EP_4_ receptors for PGE_2_ were antagonized. Elevated levels of PGE_2_ were found in cultures of normal human bronchial epithelial cells exposed to pulmonary surfactant. We conclude that prostaglandin E_2_ and its EP_2_ and EP_4_ receptors are likely involved in the relaxant effect of pulmonary surfactant in airways.

## Graphical abstract

The relaxant effect of pulmonary surfactant on airway smooth muscle (Prostaglandin E2).

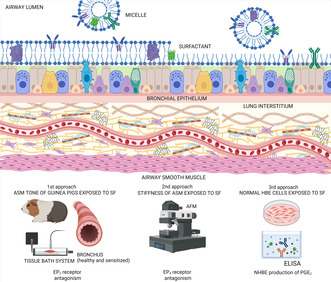

## 1 INTRODUCTION

Pulmonary surfactant is a surface‐active substance on lung epithelium composed of phospholipids (PLs), neutral lipids, and surfactant‐associated proteins (SP‐A, SP‐B, SP‐C, SP‐D). Surfactant has essential roles in lung physiology, as evidenced by observations that its dysfunction or deficiency contributes to the pathophysiology (Goerke & Clements, 2011) of several life‐threatening respiratory diseases such as respiratory distress syndrome (Avery & Mead, 1959; Matthay et al., 2019), lung oedema (Islam et al., 2022), pneumonia (Günther et al., 2012), cystic fibrosis (Griese et al., 1997), bronchial asthma (Enhorning et al., 2000; Hohlfeld, 2002), and chronic obstructive pulmonary disease (COPD) (Obeidat et al., 2017). Airway surfactant originates mainly from the alveoli where most of it is synthesized and secreted by alveolar type II cells. During expiration, alveolar surfactant becomes extruded into adjacent conducting airways (Bailey & Veldhuizen, 2005). Pulmonary surfactant lowers surface tension at the air‐liquid interface and prevents the collapse of bronchioles and alveoli, forms a barrier against inhaled particles, serves as a frontline in lung host defense (Wright, 2003), and relaxes airway smooth muscle upon contact with epithelium (Calkovska et al., 2015; Koetzler et al., 2006; Topercerova et al., 2019). Several studies indicate the potential of surfactant to reduce bronchial constriction (Becher, 1985; Hohlfeld et al., 1997) and improve lung functions in asthmatics (Babu et al., 2003; Kurashima et al., 1997; Liu et al., 1996). The patients with severe asthma (5%–12% of the total asthmatic population) have frequent severe and serious exacerbations, or airflow limitation, despite treatment with high‐dose inhaled corticosteroids (ICS) and long‐acting β_2_‐agonists (LABAs), leukotriene modifiers, or theophylline (Calhoun & Chupp, 2022). In 2019, asthma led to almost 1000 deaths every day and affected 262 million people worldwide (The Lancet, 2020). To reduce the clinical and economic burden, new treatment approaches and cost‐effective biologic treatments are required (Zeiger et al., 2016). So far, the exact pharmacological relaxant mechanism of surfactant remains largely unknown, but there is some evidence that the relaxant effect is partially dependent on prostanoid synthesis and requires intact epithelium (Koetzler et al., 2006). Participation of the ATP‐dependent potassium channels, the cAMP‐regulated epithelial chloride channels (CFTR) and nitric oxide (NO) have not been confirmed (Calkovska et al., 2015). The release of arachidonic acid from cell membrane PLs, through the action of a family of phospholipases (e.g., PLA_2_), can result in the production of a wide variety of prostanoids (Barnes, 2002). Arachidonic acid is converted by cyclooxygenase (COX) to prostaglandin H_2_, a substrate for production of various prostaglandins. Prostaglandin E_2_ is produced in significant amounts by airway epithelial cells under physiological (Pavord & Tattersfield, 1995) and pathophysiological circumstances and is a potent airway smooth muscle relaxant and inhibitor of the bronchoconstrictor response (Barnes, 2002; Konya et al., 2013). The EP_4_ receptor is one of the four receptors specific for PGE_2_ (EP_1–4_) and belongs to the superfamily of G‐protein coupled receptors. PGE_2_ activates the EP_4_ receptor on human ASM and the EP_2_ receptor on guinea pig ASM (Säfholm et al., 2013) and associates with Gαs‐subunit leading to increased production of cAMP and smooth muscle relaxation (Kim et al., 2021). Therefore, the aim of the study was to test by in vitro methods of organ bath and atomic force microscopy (AFM) whether the relaxant effect of pulmonary surfactant on airway smooth muscle is mediated through prostaglandin E_2_. This effect was investigated first by antagonizing EP_2_ receptor for PGE_2_ in ASM of guinea pigs and second, by blocking EP_4_ receptor in human ASM cells.

## 2 MATERIALS AND METHODS

### 2.1 Tracheal and lung tissue organ bath experiments

The design of the study was approved by the local Ethics Committee of the Jessenius Faculty of Medicine, Comenius University in Martin (permission no. EK 46/2018) and the State Veterinary and Food Administration of the Slovak Republic.

### 2.2 Experimental animals

Twenty‐four male young adult guinea pigs (Dunkin‐Hartley, 300–350 g of b.w.) were obtained from the breeding station Velaz, Ltd. (Prague, Czech Republic) and kept in the Faculty Animal Facility for 5 days quarantine and further 21‐day challenge period at standard conditions (12 h light/dark cycle, temperature 20°C–24°C, humidity 55 ± 10%, and food (Altromin 3123; Altromin Spezialfutter GmbH & Co. KG, Lage, Germany) and water at their disposal). The animals were sacrificed by intraperitoneal injection of anesthetics Zoletil at the dose 150 mg/kg of b.w. (Virbac SA, Carros, France) combined with Xylariem at the dose of 50 mg/kg of b.w. (Ecuphar N.V., Oostkamp, Belgium) 24 h after the last inhalation of challenge protocol and used for in vitro measurements.

### 2.3 Chemicals

Porcine pulmonary surfactant for clinical use poractant alfa (Curosurf®) was obtained from Chiesi Farmaceutici, Parma, Italy. The methacholine chloride, indomethacin, adjuvant aluminium hydroxide, dimethyl sulfoxide (DMSO), and albumin from chicken egg white (ovalbumin) were purchased from the Merck KGaA (Darmstadt, Germany). The EP_2_‐receptor antagonist PF‐04418948 was purchased from the Cayman Chemicals (Ann Arbor, Michigan, United States). DMSO was used as a solvent for stock solutions of PF‐04418948 and indomethacin with final concentration 0.01%–0.1% in 20 mL organ chamber. The sterile saline (0.9% NaCl) was used for inhalation solutions and bronchoalveolar lavage procedure and Aqua pro injectione was used as a solvent for ovalbumin injection and methacholine solution (both from B. Braun company Melsungen AG, Germany). Krebs–Henseleit solution was prepared fresh daily from the NaCl 110.0, KCl 4.8, CaCl_2_ 2.35, MgSO_4_ 1.2, KH_2_PO_4_ 1.2, NaHCO_3_ 25.0, Glucose 10.0 mmoL/L in distilled water (purchased from Centralchem s.r.o., Bratislava, Slovakia) (Table S1).

### 2.4 The design of study

The animals were randomly divided into the two groups: control (*n* = 9) and ovalbumin (ova challenged) (*n* = 11). Sample size was consulted with the authors of applied challenge protocol (Mokry et al., 2008). The challenged animals received ovalbumin injection (1% ovalbumin with 0.1% adjuvant in 1 mL of sterile water) on day 1 (0.5 mL intraperitoneally and 0.5 mL subcutaneously) and day 3 (1 mL intraperitoneally). Inhalation with 1% ovalbumin in sterile saline aerosol was performed on the days 14 and 21. Twenty‐four hours after the last inhalation with ovalbumin (challenge) the animals were sacrificed by lethal dose of anesthetics. The same protocol was used for the control group, except the ovalbumin was substituted with sterile water for injection and saline for inhalation. Tracheal and lung tissues containing airway smooth muscle were excised *postmortem*. Trachea was purified from surrounding tissues and cut on both sides into 15 mm long strips and cut in the longitudinal axis opposite to the smooth muscle layer. Lung tissue strips were cut from the middle lobe of the right lung (2 mm × 2 mm × 15 mm). Tracheal and lung strips were then connected to the threads and mounted on plastic holder and immediately placed into the organ chamber for measurement of airway reactivity. Each control tracheal and lung tissue strip was precontracted with methacholine (10^−6^ M) until stable contraction was reached and then exposed to exogenous surfactant Curosurf (1 mg of PLs/mL). After proper washout and return of the tissue tension back to the baseline values the experiment was repeated in the presence of indomethacin, COX inhibitor, to study relaxation mechanism of surfactant and prostanoid synthesis dependence. The tissues were pre‐incubated with indomethacin at 10^−5^ M concentration for 30 min and indomethacin was further present when methacholine or surfactant added in the chamber. Another control tissue strip from the same animal was examined with the same protocol; however, in the presence and absence of EP_2_‐receptor antagonist, PF‐04418948, to study the relaxation mechanism of surfactant and involvement of EP_2_ receptor. The tissues were pre‐incubated with PF‐04418948 at 10^−5^ M concentration for 45 min and EP_2_‐antagonist was further present when methacholine or surfactant added in the chamber. The third tracheal and lung tissue strip taken from the same animal was precontracted with cumulative doses of methacholine chloride (10^−8^–10^−3^ M) and served as a control to the ovalbumin challenged group for tissue sensitivity assessment. Each ova challenged animal provided two tracheal and lung tissues. One tissue strip was precontracted with cumulative doses of methacholine (10^−8^–10^−3^ M) and served as a marker of increased airway hyperresponsiveness after ovalbumin challenge. The other tissue strip was precontracted with a single dose of methacholine (10^−6^ M) and exposed to surfactant (1 mg PLs/mL) for testing its relaxant effect in hyperresponsive airways. Tracheal and lung tissues from four animals were used for the investigation of the effect of surfactant on methacholine precontraction (tissues from the same four animals were exposed to sterile saline and served as a control group).

### 2.5 Airway reactivity in vitro measurements

The isometric tension of tracheal and lung tissue strips was evaluated in the tissue organ bath system with eight chambers (Experimetria, Hungary) with SPEL Advanced ISO v3.2 software installed. As mentioned above, tissue strips were carefully mounted in between the hooks of the holder and placed in 20 mL organ chambers filled with Krebs–Henseleit buffer. Tissue viability was maintained at constant temperature 37°C and physiological pH 7.4 of the buffer gassed with a pneumoxide (95% O_2_ and 5% CO_2_). The strips were tied with the threads to a tensiometer connected to an amplifier and tension was recorded. Tissue strips were allowed to equilibrate for 30 min at 4 g tension and another 30 min at 2 g resting tension (baseline value; on *y* axes of the figures displayed as zero) before being exposed to agonists, while rinsed every 10 min with fresh buffer.

### 2.6 Airway smooth muscle cell culture

**FIGURE 1:**
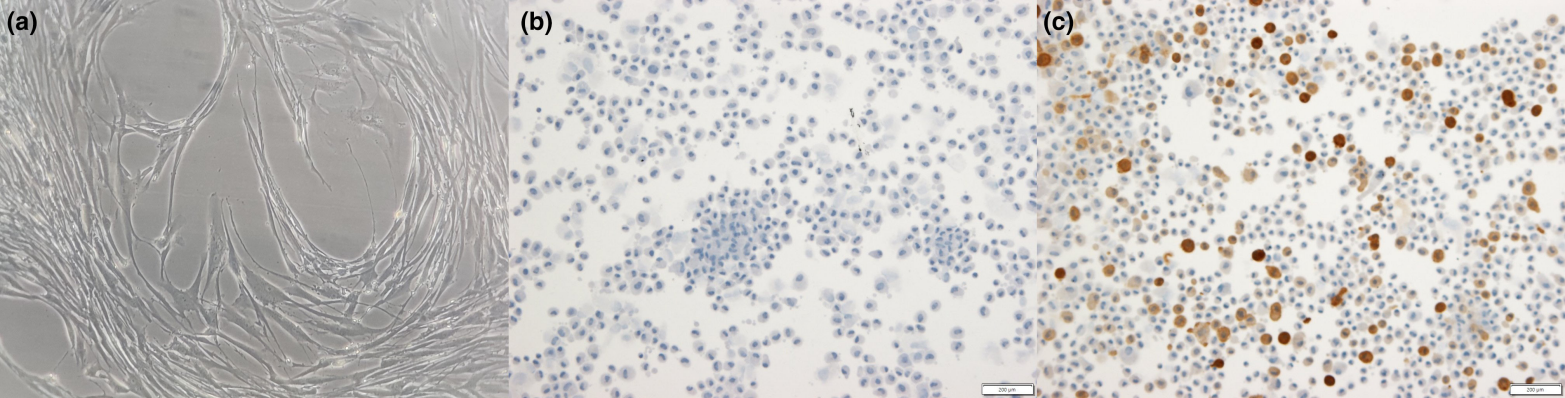
Primary human bronchial airway smooth muscle cells. (a) Cells of fifth passage of donor no. 1 growing in T75 flask (10× objective). (b) Negative control of ASM cells stained for smooth muscle α‐Actin (primary mouse alpha‐smooth muscle actin monoclonal antibody omitted) (10× objective). (c) Positive control of ASM cells stained for smooth muscle α‐Actin (primary and biotinylated goat anti‐mouse IgG secondary antibody included) (10× objective). Cells stained by brown chromogen DAB (3,3’‐Diaminobenzidine tetrahydrochloride). Specificity of the antibodies declared in Supplement document.

Primary human airway smooth muscle cells provided by Dr. Margaret Kelly and Dr. David Proud were isolated and cultured according to previously described methods (Shariff et al., 2017). The cells were obtained from two donors by microdissection (donor no. 1: 30 years old male of Hispanic race; donor no. 2: 62 years old female of Caucasian race). Protocols for obtaining cells were approved by the University of Calgary (Calgary, Alberta, Canada) Conjoint Health Ethics Board. ASM cells were isolated and enzymatically dissociated with protease and cultured in Dulbecco's modified Eagle's medium (DMEM) supplemented with 10% fetal bovine serum and antibiotics. Cell culture reagents were purchased from Thermofisher Scientific Inc. (Mississauga, Ontario, Canada). Cells of passage 2 and 3 were thawed and cultured in submersion in 75cm^2^ flasks (Corning Inc., NY, USA) in DMEM medium containing 10% fetal bovine serum and 1% penicillin–streptomycin‐amphotericin B supplement at 37°C in humidified air containing 5% CO_2_ until 70% confluence. The medium was changed every 48 h, and confluent cells were washed (Hanks' Balanced Salt Solution) and passaged with 0.25% Trypsin–EDTA every 10–12 days until passage 6. The cultured cells were identified as smooth muscle cells as elongated and spindle shaped, with the typical hill‐and‐valley appearance when examined by light microscopy, and positive immunohistochemistry staining for smooth muscle α‐Actin using cytospin method (Figure 1) (Table S2).

Prior to AFM experiments, cells were incubated for 24 h in serum‐free DMEM medium. The day of experiment, serum‐free medium was changed for a fresh medium equilibrated in CO_2_ incubator.

### 2.7 ASM cell stiffness measured by atomic force microscope

**FIGURE 2:**
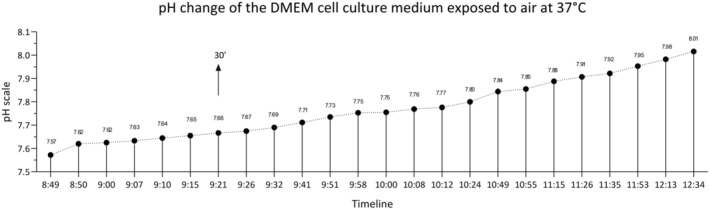
pH change of the cell culture medium DMEM exposed to air and heated to 37°C. DMEM in plastic tube was immersed in 37°C heated water bath and pH value was periodically read by pH electrode.

Cell stiffness was measured in real time using the single cell force spectroscopy (SCFS) mode of an atomic force microscope according to Amrein & Stamov (2019). Measurements were performed with a NanoWizard IV AFM atomic force microscope (JPK Instruments, Berlin, Germany). The cantilevers used in the experiments were made of silicon nitride and coated with a layer of gold. The cantilever spheroid had a diameter of 5.3 μm (AppNano, Inc., Mountain View, California, USA), a nominal spring constant of *k* = 0.035 N/m, and a resonance frequency of 20 kHz. Petri dishes (ibidi GmbH, Gräfelfing, Germany) with ASM cells were mounted on the microscope stage (preheated at 37°C). Following calibration, AFM cantilever operated in contact mode of advanced single cell‐force spectroscopy mode and scanned cell surface at a speed of 10 μm/s at a load force (setpoint) of 1 nN. The cantilever height and deflection data during the contact with cell were collected and processed using JPK software system (Santa Barbara, CA, USA) to provide force‐distance curves. The recorded force curves were analyzed using the Hertzian model to calculate the cell stiffness and expressed as Young's modulus value (Pa), which defines relationship between applied force onto the cell and its deformation (stress/strain). Stiffness was recorded after 5‐min incubation of ASM cells with cholinergic muscarinic receptor agonist methacholine chloride at concentration 10^−4^ M (MilliporeSigma Canada Co., Oakville, ON, Canada), the smooth muscle relaxant prostaglandin PGE_2_ at concentration 10^−7^ M (MilliporeSigma Canada Co., Oakville, ON, Canada), exogenous bovine lung surfactant BLES at 2 μL/mL with 0.05 mg of PLs/mL (BLES Biochemical, London, ON, Canada) and sterile water. The stiffness of ASM cells after pulmonary surfactant was recorded in the presence of the EP_4_ receptor blocker for PGE_2_—ONO‐AE3‐208 at a concentration of 10^−6^ M (MilliporeSigma Canada Co., Oakville, ON, Canada) after 45′ incubation (Table S3). Minimum of three cells were tested for each agonist. Single cell grown in monolayer was contacted at nucleus and cytoplasm area by AFM cantilever before and after the agonist addition. At least, 64 points were contacted on nucleus and cytoplasm area of each cell respectively during stiffness measurement. First, the baseline stiffness was recorded before adding the agonists (methacholine, PGE_2_, BLES surfactant, and sterile water) into the cell medium. Cell points contacted after the agonist addition to the cells come from approximately the same cell area, but they are not identical with the points contacted during baseline measurement (Figures [Link], [Link] and S8). This is due to the movement of the AFM cantilever during agonist injection. Micro‐dish with cells was outside the CO_2_ incubator on preheated AFM microscopic stage (37°C) for 20–30 min during the measurement. Change of the medium pH was tested for stability in separate experiment. After 30 min, pH of pure DMEM medium exposed to air at 37°C increased from 7.57 to 7.66 on pH scale (Figure 2).

### 2.8 Normal human bronchial epithelial cells

**FIGURE 3:**
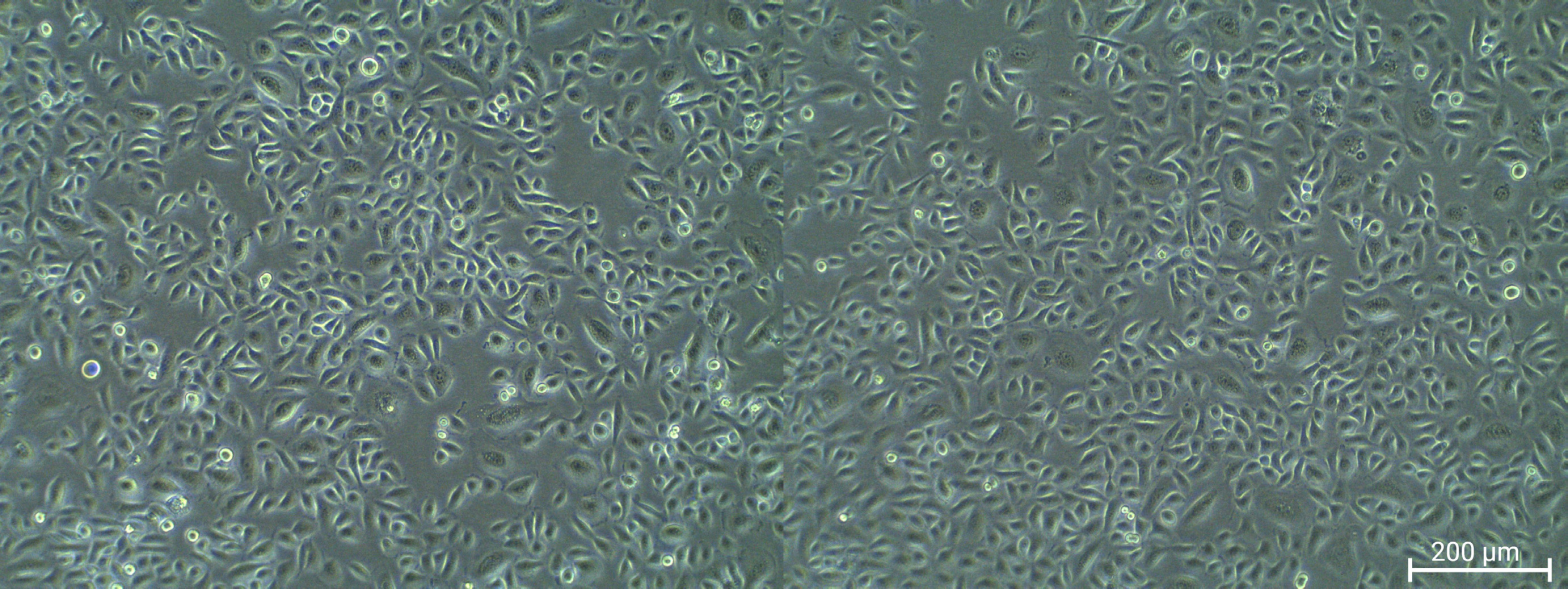
Primary normal human bronchial epithelial cells in submersion culture after 4 days prior surfactant stimulation (10× objective).

Normal human bronchial epithelial (NHBE) cells grown in submersion culture were incubated with surfactant for 24 h. After incubation, cell culture medium of the cells was collected and analyzed for levels of PGE_2_. Primary human bronchial epithelial cells obtained from Lonza (Walkersville, MD, USA; catalog no. CC‐2540) were cultured according to manufacturer's instructions. All cell culture reagents were purchased from Lonza (Walkersville, MD, USA). Cells were thawed and cultured in submersion in 75cm^2^ flasks in bronchial epithelial cell growth medium (BEGM) at 37°C in humidified air containing 5% CO_2_ until 80% confluence (80% reached in approximately 4 days). The medium was changed every 48 h, and confluent cells were passaged with 0.25% Trypsin–EDTA every 4 days (Figure 3). After passaging, cells were further seeded in 75cm^2^ flasks to obtain other passages and/or seeded on 6‐well plate for surfactant incubation (Sarstedt, Nümbrecht, Germany) at 60%–80% confluence (Table S4).

Prior to surfactant stimulation, cells in 6‐well plates were incubated for 24 h in bronchial epithelial cell basal medium (BEBM) without supplements. The day of stimulation, negative control received fresh BEBM and BLES group received fresh BEBM supplemented with 1 mg of PLs/mL of BLES surfactant. Cell culture media were collected 24 h after stimulation, stored at −80°C until assayed by ELISA analysis for PGE_2_ concentration. NHBE cells were tested as a pilot experiment and therefore obtained from one donor (51 years old male of Hispanic race). Cell cultures of four different passages were used to obtain four biological replicates. If the primary cells crossed the doubling limit (15 population doublings guaranteed by Lonza manufacturer), a typical apoptosis sign of cell membrane blebbing occurred and these cells were not included in the experiments.

### 2.9 Competitive enzyme linked immunosorbent assay (ELISA) for PGE_2_ quantification

Levels of prostaglandin E_2_ were quantified (pg/mL) in cell culture media collected after BLES surfactant incubation with the NHBE cells for 24 h according to manufacturer’s instructions (Cayman Chemical Company, Ann Arbor, Michigan, USA, cat. no. 514010). Samples were run in triplicates.

### 2.10 Statistical analysis

The data collected from tissue organ bath system were analyzed with paired Student’s *t*‐test to compare the tension at baseline to the tension after surfactant and the change in tension after surfactant before and after antagonists. In these “before and after” measurements the responses were paired since coming from within the same tissue. If data failed normality test (Shapiro–Wilk) and the data distribution did not appear to be sampled from a Gaussian distribution based on QQ normality plot, Wilcoxon matched‐pair signed rank test for paired data and non‐parametric distribution was used. Levels of PGE_2_ in cell culture media from NHBE cells exposed to pulmonary surfactant were analyzed with paired Student's *t*‐test. To confirm increased airway hyperresponsiveness after ovalbumin challenge, the EC_50_ values (half maximal effective concentration) were derived from fitting dose–response stimulation curve using nonlinear regression and compared by unpaired Student's *t*‐test. Results are expressed as mean ± standard deviation (SD) or show all data points (min to max with median line). The data were processed and statistically evaluated in the software GraphPad Prism version 8.0.1 (GraphPad Software, USA). To compare the effect of surfactant on muscle tension of control and ovalbumin‐challenged animals, robust and resistant estimates of location of the distribution of resistance were obtained by the marginal mean (MM) method. Using the MM fit, confidence intervals and *p*‐value for the test of the null hypothesis of equality of population MM estimates of location of the distribution of resistance in control and ovalbumin‐challenged populations were obtained by the MMs contrast. MM method was performed in the R environment (R Foundation for Statistical Computing, Vienna, Austria) version 4.0.5.

The linear mixed models (LMMs) were employed to compare airway smooth muscle cell stiffness before and after agonist exposure accounting for the repeated measures. We used LMM as a powerful tool when analyzing atomic force microscope data with repeated measurements from the same statistical units. It handles the correlations between observations (by incorporating random effects for random intercepts and slopes), accounts for inherent similarities within grouped data and allows each subject or unit to exhibit unique characteristics over time. They are particularly adept at dealing with missing data, assuming it is missing at random, and they enhance the statistical power of the analysis by utilizing all available data points. Additionally, these models can handle complex variance–covariance structures and time‐dependent covariates, making them highly flexible and capable of modeling different variance components and covariance structures. Although the sample size is limited, we had a large number of repeated measurements and that can significantly enhance the quality and reliability of the statistical analysis—it improves estimation accuracy and provides deeper insights into within‐subject variability. Furthermore, a larger number of repeated measurements per subject helps mitigate issues with missing data and non‐normality, enabling more robust and sophisticated analyses. It is especially valuable in research where increasing the overall number of subjects is not feasible. The analysis was conducted using R program (R Foundation for Statistical Computing, Vienna, Austria, version 4.3.2) and it reveals statistically significant effect of agonists on cell stiffness. Descriptive statistics of cells measured by AFM for Young's Modulus evaluation (SD, standard error of mean (SEM), mean, and median are shown in the Tables S5–S12). For all data, a *p*‐value less than 0.05 was considered statistically significant (*p* < 0.05).

## 3 RESULTS

### 3.1 The effect of ovalbumin on tracheal and lung tissue tension—Model of bronchial hyperresponsiveness

**FIGURE 4:**
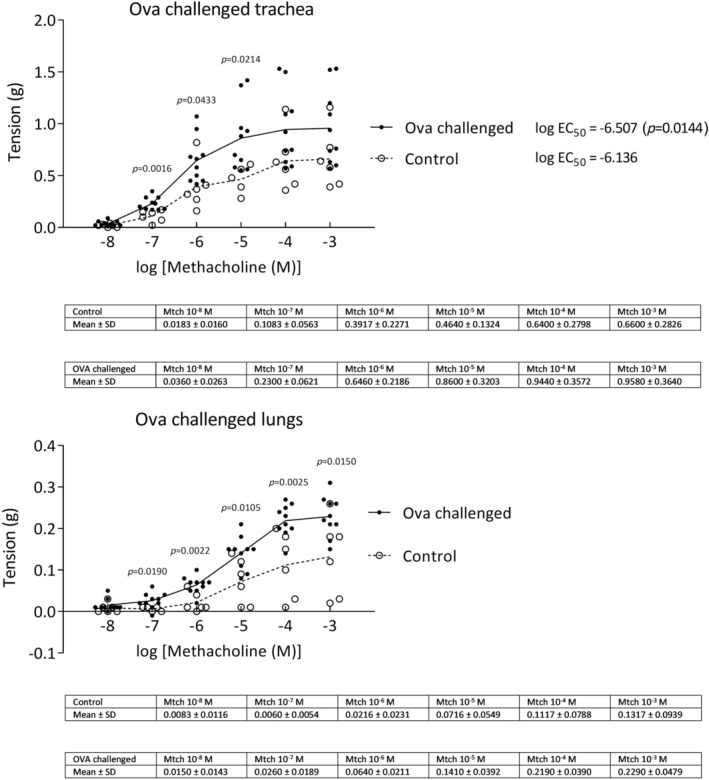
Tension of tracheal and lung tissue strips precontracted with cumulative doses of methacholine (Mtch 10^−8^–10^−3^ M) after 21‐day ovalbumin challenge (ova challenged). Control *n* = 6; ova challenged *n* = 11. Data on the figure are shown as individual replicates with means connected and mean ± SD are shown in the table below. Log EC_50_ values for control and ova challenged groups made by non‐linear regression (dose–response stimulation curve) and compared by two‐tailed unpaired Student's *t*‐test. Comparisons of each cumulative dose of methacholine between control and ova challenged group made by unpaired Student's *t*‐test.

Ovalbumin was used to establish the model of bronchial hyperresponsiveness. Tracheal tissues of challenged animals were found hyperresponsive after methacholine challenge compared to control group (potency of methacholine was significantly increased in challenged tracheal tissues based on log EC_50_ values). Methacholine at the doses of 10^−7^—10^−5^ M increased tension of tracheal tissues and at 10^−7^—10^−3^ M increased tension of lung tissues compared to control group (Figure 4).

### 3.2 The effect of surfactant on tension of ovalbumin challenged airway tissues

**FIGURE 5:**
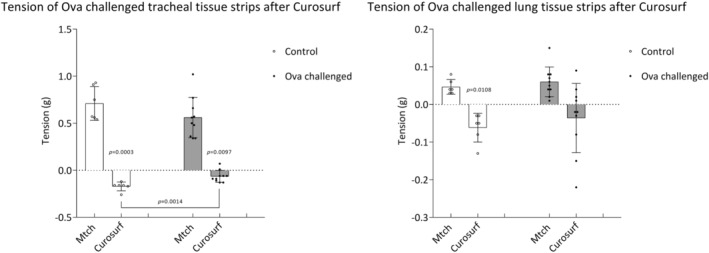
The effect of Curosurf on tension of tracheal and lung tissue strips precontracted with methacholine (Mtch) (10^−6^ M) after 21‐day ovalbumin challenge (ova challenged). Control *n* = 6; ova challenged n = 10. Data are shown as mean ± SD. Baseline value is set to zero on *y* axis (intermittent line) and represents +2 g resting tension before adding Mtch and represents the level of stable contraction before adding Curosurf. Therefore, an increase of tension is displayed as a positive change and decrease of tension is displayed as a negative change. Comparisons within the groups made by two‐tailed paired Student's *t*‐test (tension at baseline compared to tension after surfactant Curosurf). Comparison between control Curosurf and ova challenged Curosurf in trachea was made by Marginal mean (MM) method for independent samples.

Tissues with airway smooth muscle from ovalbumin challenged animals were precontracted with methacholine and exposed to pulmonary surfactant Curosurf in tissue organ chambers. Curosurf at 1 mg PLs/mL reduced the tension of precontracted tracheal and lung strips in control groups. Curosurf reduced the tension of ova challenged tracheal tissue strip and the difference between control and ova challenged trachea was significant (Figure 5).

### 3.3 The effect of surfactant on tension of normal airway tissues after inhibition of prostanoid synthesis

**FIGURE 6:**
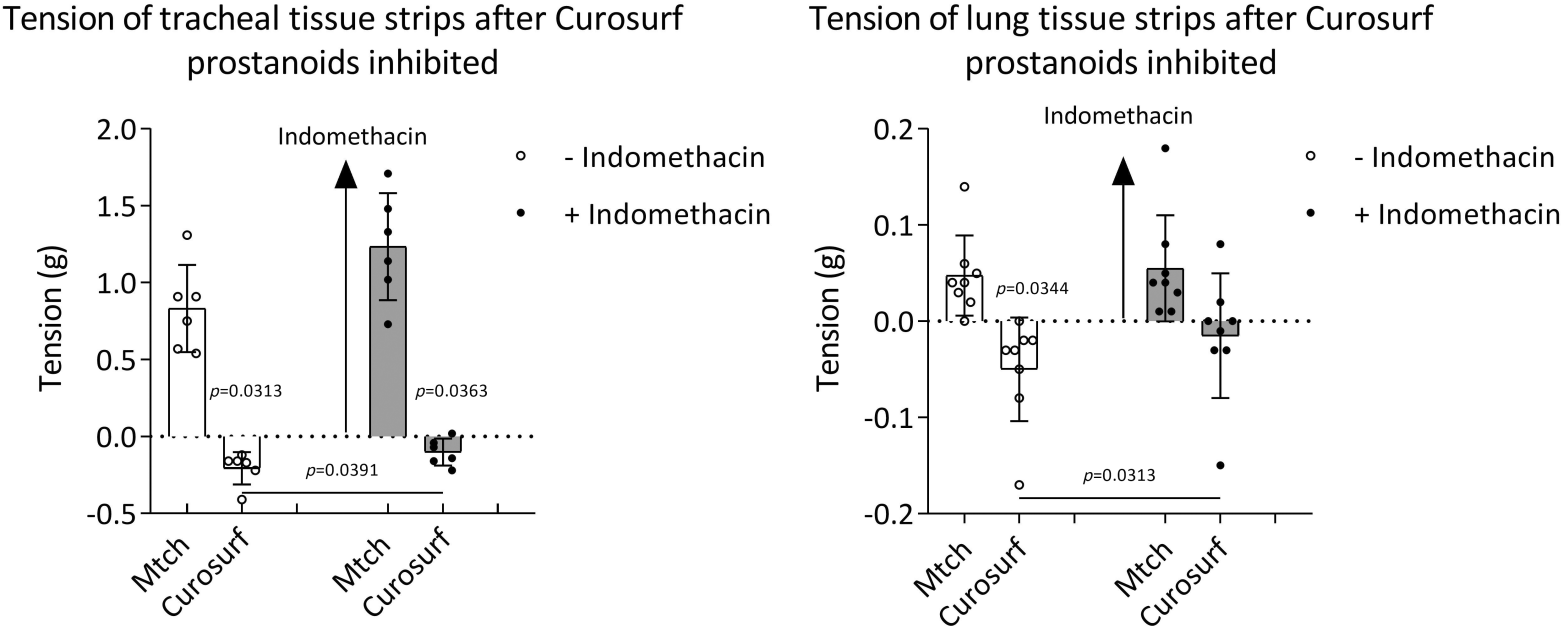
The effect of Curosurf in presence of prostanoid synthesis inhibitor—indomethacin on tension of tracheal and lung tissue strips precontracted with methacholine (Mtch) (10^−6^ M). Tracheal tissue strips *n* = 6; lung tissue strips *n* = 8. (−) without indomethacin; (+) with indomethacin. Data are shown as mean ± SD. Baseline value is set to zero on *y* axis (intermittent line) and represents +2 g resting tension before adding Mtch and represents the level of stable contraction before adding Curosurf. Therefore, an increase of tension is displayed as a positive change and decrease of tension is displayed as a negative change. Comparisons made by two‐tailed paired Student's *t*‐test or two‐tailed Wilcoxon matched‐pair signed rank test.

The relaxant effect of surfactant was tested in the presence of indomethacin, inhibitor of prostanoid synthesis. Curosurf reduced tension of precontracted tracheal tissue strips in and out of indomethacin presence. Collected data revealed a significant difference between Curosurf effect on tracheal tension without and with indomethacin. Curosurf reduced lung tension without indomethacin. The difference between Curosurf effect on lung strip tension without and in the presence of indomethacin was significant (Figure 6).

### 3.4 The effect of surfactant on tension of normal airway tissues after EP_2_ ‐receptor antagonism

**FIGURE 7:**
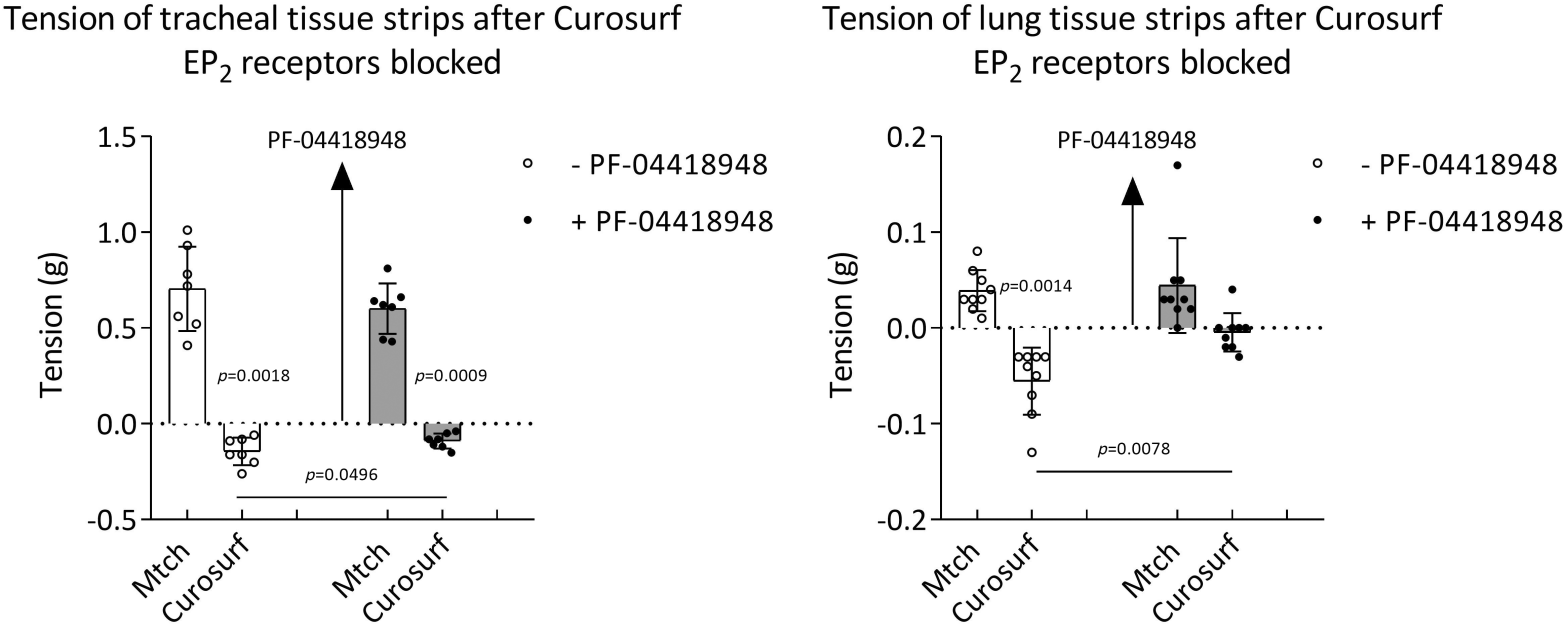
The effect of Curosurf after EP_2_‐receptor antagonist PF‐04418948 on tension of tracheal and lung tissue strips precontracted with methacholine (Mtch) (10^−6^ M). Tracheal tissue strips *n* = 7; lung tissue strips *n* = 9. (−) without PF‐04418948; (+) with PF‐04418948. Data are shown as mean ± SD. Baseline value is set to zero on *y* axis (intermittent line) and represents +2 g resting tension before adding Mtch and represents the level of stable contraction before adding Curosurf. Therefore, an increase of tension is displayed as a positive change and decrease of tension is displayed as a negative change. Comparisons made by two‐tailed paired Student's *t*‐test or two‐tailed Wilcoxon matched‐pair signed rank test.

The relaxant effect of surfactant was tested after blockade of EP_2_ receptors, normally mediating relaxant effect of PGE_2_, with antagonist PF‐04418948. Curosurf reduced tracheal tension in both conditions, with and without EP_2_ receptor antagonist. The difference between Curosurf effect on tracheal tension without and in the presence of PF‐04418948 was significant. Curosurf reduced tension of lung tissue strip without PF‐04418948; however, the ability of Curosurf to reduce lung tension was diminished in the presence of PF‐04418948. The difference between Curosurf effect on lung tension without and in the presence of EP_2_ receptor blocker was significant (Figure 7).

**FIGURE 8:**
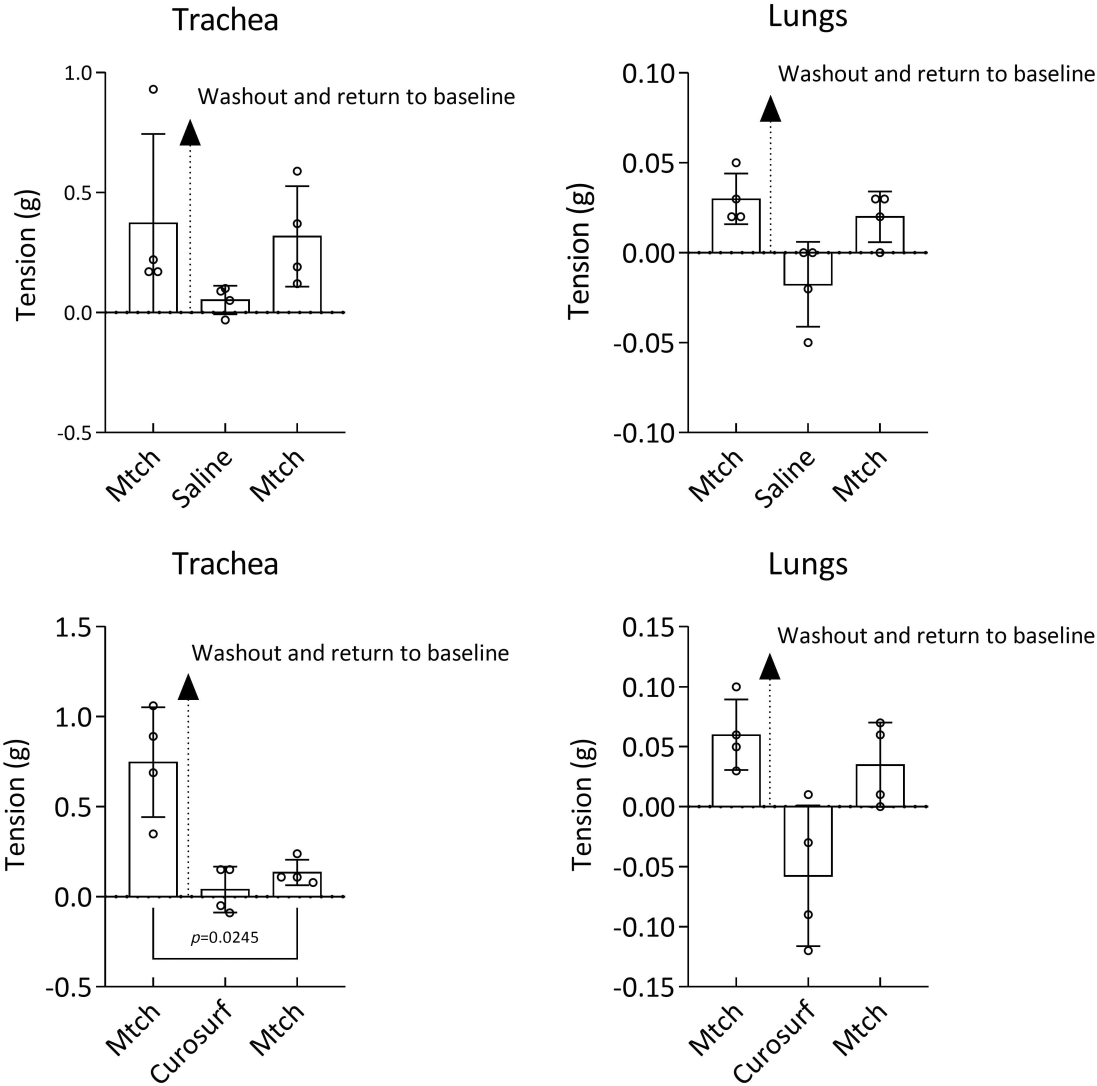
The effect of Curosurf on methacholine precontraction (Mtch; 10^−6^ M) of tracheal and lung tissue strips (*n* = 4). Data are shown as mean ± SD. Baseline value is set to zero on *y* axis (intermittent line) and represents +2 g resting tension before adding Mtch, sterile saline or Curosurf. Therefore, an increase of tension is displayed as a positive change and decrease of tension is displayed as a negative change. Comparison made by two‐tailed paired Student's *t*‐test.

We performed another experiment, where tension at methacholine precontraction was observed before and after surfactant. First, we precontracted the tissues with a single dose of methacholine and after stable contraction was reached, we removed the constrictor from the chamber in order to prevent suppressing effect on the following methacholine precontraction (washout arrow in the Figure 8). After tension of the tissues reached the baseline values, we added surfactant and precontracted the tissues with methacholine again. The response of tracheal tissues to methacholine was significantly lowered after surfactant compared to the first precontraction. The effect of sterile saline on tissue tone was not significant (Figure 8).

### 3.5 Atomic force microscope recordings of airway smooth muscle cell stiffness

Currently, measurement of isometric tension of animal excised muscle fibers in a tissue bath is used to test the relaxant effect of surfactant. The study proposes an alternative setup, based on cultures of human airway smooth muscle cells and measure of the contractile state from the cell stiffness alternatively to the tissue organ bath method. The tissue organ bath method requires tracheal and lung tissue strips from multiple animals and can be time‐consuming and prone to smooth muscle layer damage and tissue viability may vary. The proposed method uses AFM and SCFS to measure cell stiffness (Thomas et al., 2013), which reflects the cytoskeletal structure (Elson, 1988) and myosin activity (Schäfer & Radmacher, 2005). The study on magnetic twisting cytometry reported that the activation of the contractile apparatus is reflected in a stiffening of the airway smooth muscle cells, where increasing doses of bronchodilator agonists that elevate intracellular cAMP and cGMP levels led to a decrease in stiffness (An et al., 2002). The study took an advantage of these observations but recorded the stiffening and softening of the ASM cells by SCFS. This approach is faster, requires minimal sample size, may serve as tool to establish the muscle cell responsiveness and drug response, and can potentially be used for high‐throughput drug screening. We hypothesize that cell stiffness as measured by SCFS adequately reveals the contractile state of the ASM cell, that is, the method reproduces results find in the organ bath. We further hypothesize that blockade of PGE_2_ membrane receptors prevents decrease in stiffness and works antagonistically to pulmonary surfactant's relaxant effect. The primary cell cultures of human airway smooth muscle cells were used in the AFM experiments and direct effect of surfactant on cell stiffness was tested. First, the sensitivity of the assay was established by measuring cell stiffness before and during induction of contraction by methacholine and relaxation by PGE_2_. Next, the effect of surfactant was tested before and after EP_4_‐recetor antagonism. The effect of sterile water as non‐surfactant treatment was tested and no significant effect on cell stiffness was detected (Figure S7).

### 3.6 The effect of contractile agonist methacholine chloride on airway smooth muscle cell stiffness

**FIGURE 9:**
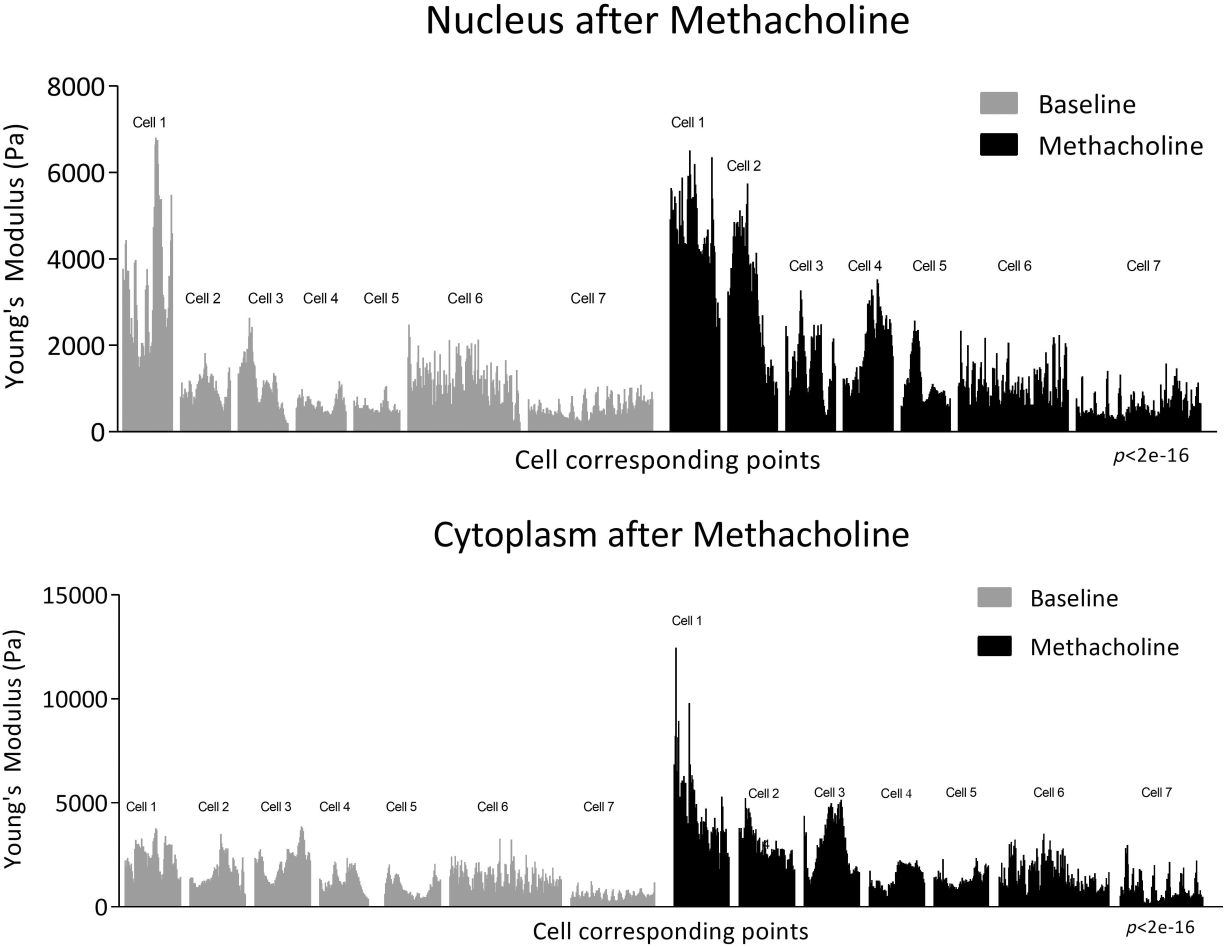
Airway smooth muscle cell stiffness of nucleus and cytoplasm after methacholine. Stiffness is expressed as Young's Modulus (Pa). Stiffness of each cell (*n* = 7 cells) was recorded at minimum of 56 cell points at baseline and after 5‐min incubation with methacholine (10^−4^ M). Cell corresponding points measured at baseline and after methacholine were taken from the same cell, but the points are not identical. The all data points are displayed. Comparison made by LMM and the Type III Wald *F* test.

Incubation of airway smooth muscle cells with the cholinergic agonist methacholine chloride resulted in an observable increase in the stiffness of both the nucleus and cytoplasm, as illustrated in Figure 9. The LMM was applied to analyze repeated measurements of nucleus and cytoplasm values at baseline and after methacholine across seven cells, considering both fixed and random effects. The random effects, accounting for variations within each cell, showed substantial variability (SD 1125 for nucleus and 965.3 for cytoplasm) suggesting that there are notable differences in the measurements taken within same cell, while the residual variance was also notable (SD 741 for nucleus and 851 for cytoplasm) possibly attributed to factors other than within‐cell differences. The fixed effects estimate strongly supports the existence of a significant treatment effect, indicating a significant relationship between methacholine and nucleus and cytoplasm values. Specifically, the model suggests that, on average, cell mean stiffness increases by 573.09 (standard error 42.11) after methacholine compared to baseline in nucleus and by 689.95 (standard error 51.92) in cytoplasm. The *p*‐values (<2e‐16 for nucleus and cytoplasm) from the Type III Wald *F* test reinforces the significance of this association. In summary, the LMM underscores a statistically significant association between the treatment levels (baseline and methacholine) and the cell stiffness, namely nucleus, and cytoplasm value. This highlights the substantial impact of methacholine in increasing cell stiffness, emphasizing its significance in the context of the analysis (Figure 9).

### 3.7 The effect of relaxant agonist PGE_2_ on airway smooth muscle cell stiffness

**FIGURE 10:**
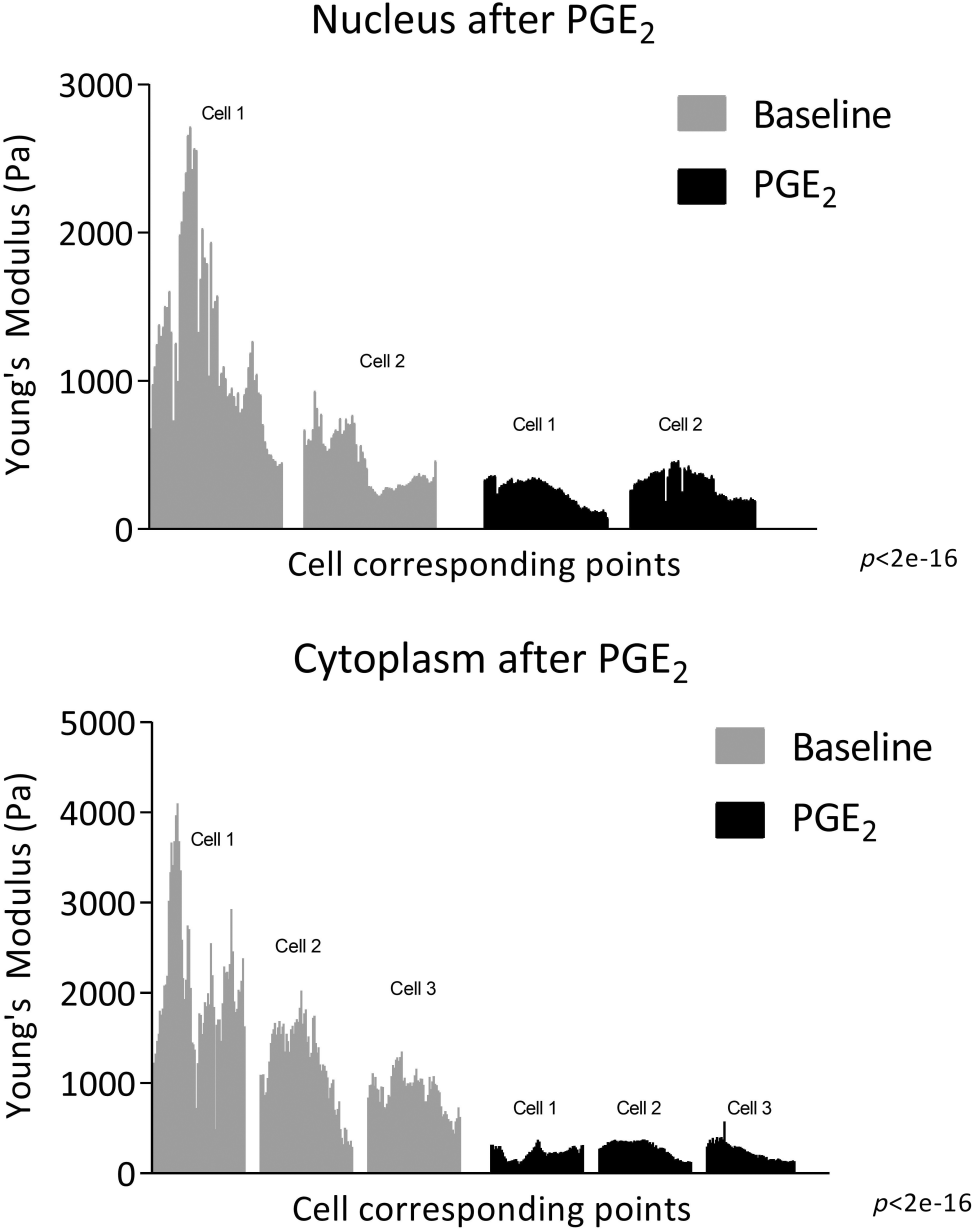
Airway smooth muscle cell stiffness of nucleus and cytoplasm after PGE_2_. Stiffness is expressed as Young's Modulus (Pa). Stiffness of each cell (*n* = 2–3 cells) was recorded at minimum of 60 cell points at baseline and after 5‐min incubation with PGE_2_ (10^−7^ M). Cell corresponding points measured at baseline and after PGE_2_ were taken from the same cell, but the points are not identical. The all data points are displayed. Comparison made by LMM and the Type III Wald *F* test.

Relaxant prostaglandin E_2_ was also used to evaluate changes in stiffness of airway smooth muscle cells. The LMM was applied to analyze repeated measurements of nucleus and cytoplasm values at baseline and after PGE_2_ across 2–3 cells, accounting for both fixed and random effect. The random effects, which capture group‐specific variation within the cells, showed substantial variability (SD 265.2 for nucleus and 304.9 for cytoplasm), while the residual variance was also notable (SD 390.5 for nucleus and 455.8 for cytoplasm). The fixed effects estimate strongly supports the existence of a significant treatment effect, indicating a significant association between PGE_2_ and nucleus and cytoplasm values. Specifically, the model suggests that, on average, cell mean stiffness decreases by 573.98 (standard error 49.51) after PGE_2_ compared to baseline in nucleus and by 1165.7 (standard error 46.7) in cytoplasm. The *p*‐values (<2e‐16 for nucleus and cytoplasm) from the Type III Wald *F* test reinforce the significance of these relationships. In summary, the LMM underscores a statistically significant association between the treatment levels (baseline and PGE_2_) and the cell stiffness, nucleus, and cytoplasm value. This highlights the substantial impact of PGE_2_ in reducing cell stiffness, emphasizing its significance in the context of the analysis (Figure 10).

### 3.8 The effect of exogenous surfactant on airway smooth muscle cell stiffness

**FIGURE 11:**
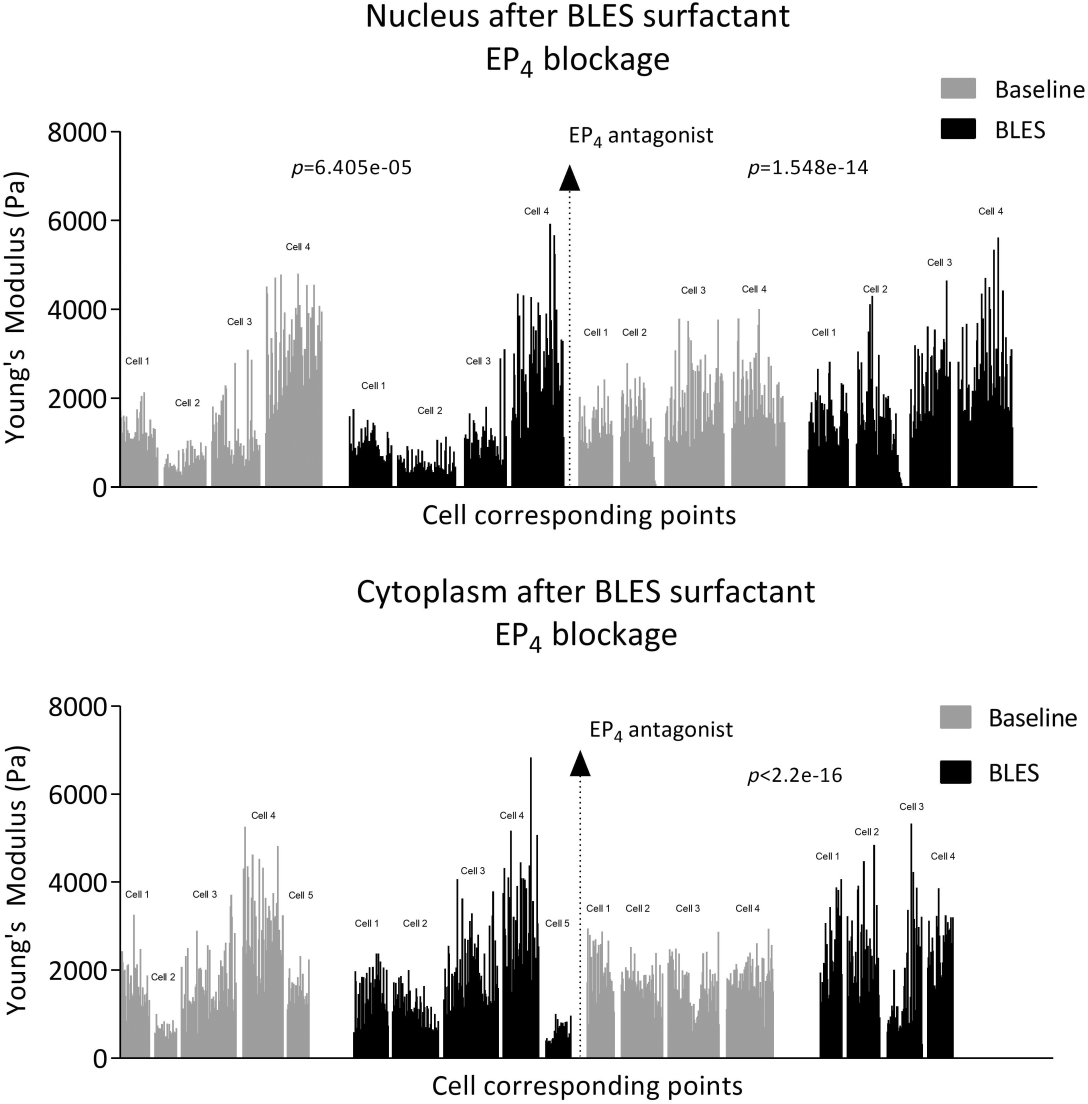
Airway smooth muscle cell stiffness of nucleus and cytoplasm after BLES surfactant before and after addition EP_4_ receptor antagonist. Stiffness is expressed as Young's Modulus (Pa). Stiffness of each cell (*n* = 4–5 cells) was recorded at minimum of 96 cell points at baseline and after 5‐min incubation with exogenous surfactant BLES (2 μL/mL with 0.05 mg of PLs/mL). After proper washout of BLES from the cell culture dishes and cell rest for 30 min in CO_2_ incubator, EP_4_ receptors were antagonized with selective antagonist ONO‐AE3‐208 at 10^−6^ M concentration during 45 min pre‐incubation in CO_2_ incubator (antagonist was further present in the medium during the measurement). Cell corresponding points were measured at baseline, after BLES and this was repeated in EP_4_ receptor antagonist presence on the same cell but the measured points are not identical due to movement of the AFM cantilever during washout steps. AFM cantilever was positioned to the approximate area of the cell after the washout (washout was performed before EP_4_ receptor addition). The all data points are displayed. Comparison made by LMM and the Type III Wald *F* test.

Airway smooth muscle cells were exposed to exogenous pulmonary surfactant and stiffness was recorded. The LMM was applied to analyze repeated measurements of nucleus and cytoplasm values at baseline and after BLES across 4–5 cells, considering both fixed and random effects. The random effects, accounting for variations within each cell, showed substantial variability (SD 988.5 for nucleus and 716.4 for cytoplasm), while the residual variance was also notable (SD 700.4 for nucleus and 725.6 for cytoplasm). The fixed effects estimate strongly supports the existence of a significant treatment effect, indicating a significant association between BLES and nucleus values. Specifically, the model suggests that, on average, cell mean stiffness decreases by 148.45 (standard error 37.03) after BLES compared to baseline. The *p*‐value (6.405e‐05) from the Type III Wald F test reinforces the significance of this association. In summary, the LMM underscores a statistically significant association between the treatment levels (baseline and BLES) and the cell stiffness, namely nucleus value. This highlights the substantial impact of BLES in reducing cell stiffness, emphasizing its significance in the context of the analysis (Figure 11). The fixed effects estimate does not indicate a significant relationship between BLES and cytoplasm values.

### 3.9 The effect of exogenous surfactant on airway smooth muscle cell stiffness after EP_4_ ‐receptor antagonism

Prostaglandin E_2_ relaxes human airway smooth muscle via activation of EP_4_ receptors. Therefore, we evaluated changes in stiffness after BLES surfactant if EP_4_ receptors antagonist ONO‐AE3‐208 was used. The LMM was also applied to analyze repeated measurements of nucleus and cytoplasm values across the same four cells at baseline and after BLES during EP_4_ receptor antagonism (Figure 11), considering both fixed and random effects. The random effects, which capture group‐specific variation within the cells, showed substantial variability (SD 394.3 for nucleus and 234 for cytoplasm), while the residual variance was also notable (SD 788.5 for nucleus and 718.1 for cytoplasm). The fixed effects estimate strongly supports the existence of a significant treatment effect, indicating a significant association between BLES and nucleus and cytoplasm values during EP_4_ receptor antagonism. Specifically, the model suggests that, on average, cell mean stiffness during EP_4_ receptor antagonism increases by 333.36 (standard error 42.91) after BLES compared to baseline in nucleus and by 355.25 (standard error 41.45) in cytoplasm. The *p*‐values (1.548e‐14 for nucleus and <2.2e‐16 for cytoplasm) from the Type III Wald *F* test reinforce the significance of these relationships. In summary, the LMM underscores a statistically significant association between the treatment levels (baseline and BLES in EP_4_ receptor antagonist presence) and the cell stiffness, namely nucleus, and cytoplasm value. This highlights the substantial impact of BLES in increasing cell stiffness during EP_4_ receptor antagonism, emphasizing its significance in the context of the analysis. Based on LMM analysis, BLES surfactant reduced cell stiffness in nuclear area if EP_4_ receptors were active; however, BLES surfactant increased cell stiffness in nuclear and cytoplasm area if EP_4_ receptors for PGE_2_ were antagonized (Figure 11).

### 3.10 The effect of surfactant on PGE_2_ production by bronchial epithelial cells

**FIGURE 12:**
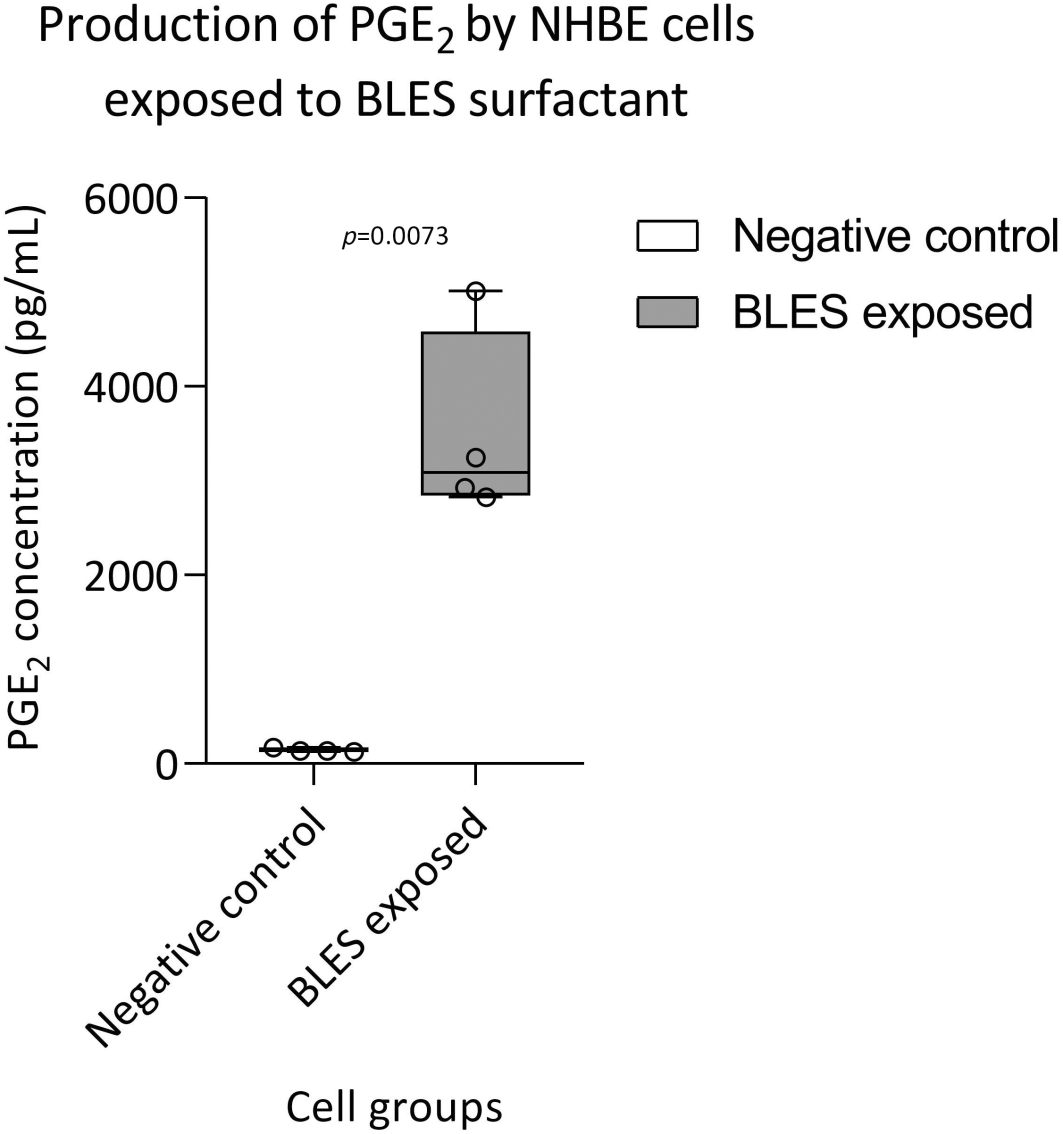
The analysis of PGE_2_ production (pg/mL) by NHBE cells after 24 h (negative control) and incubation with 1 mg PLs/mL of exogenous bovine BLES surfactant (BLES exposed). *n* = 4 biological replicates from one donor (male, 51 years, Hispanic race). All data points (min to max) are displayed with median line. Comparison made by paired Student's *t*‐test.

Normal bronchial epithelial cells that were exposed to pulmonary surfactant for 24 h at 1 mg PLs/mL were found to have increased levels of prostaglandin E_2_ in the cell culture medium compared to control cells (Figure 12). BLES surfactant solution and cell culture medium (BEBM) were tested for the presence of PGE_2_, but the concentrations were below the detection limit (data not shown).

## 4 DISCUSSION

Several studies refer that restoration of pulmonary surfactant layer in airways has beneficial effect in conditions typical for asthma or COPD. Surfactant improves mucociliary clearance and helps to relieve mucous plug (Banerjee & Puniyani, 2000), increases forced expiratory volume in 1 s (FEV_1_) (Babu et al., 2003; Kozlova et al., 2018) and airway opening pressure (Kurashima et al., 1997), keeps airway resistance low (Enhorning et al., 1995), opsonizes pathogens for their clearance from airways (Carreto‐Binaghi et al., 2016), forms a protective barrier separating bronchial air from the receptors that elicit bronchoconstriction (Hills et al., 2003), and relaxes airway smooth muscle (Calkovska et al., 2015; Koetzler et al., 2006; Topercerova et al., 2019). The relaxant effect of surfactant is epithelium‐dependent and prostanoid‐synthesis dependent (Koetzler et al., 2006), while NO, ATP‐dependent potassium channels or cAMP‐regulated epithelial chloride channels (also CFTR channels) have not been confirmed (Calkovska et al., 2015; Koetzler et al., 2006). Airway smooth muscle undergoes relaxation after exposure to surfactant, which is inhibited by bacterial lipopolysaccharide (LPS). Surfactant in combination with protective H1‐receptor antagonist mepyramine maleate and leukotriene receptor antagonist montelukast sodium reduced airway smooth muscle tone after LPS which indicates leukotriene and histamine H1‐receptor involvement in contractile mechanisms of the airways exposed to LPS (Topercerova et al., 2019).

Our research follows these findings and tends to investigate the poorly known mechanism of surfactant relaxation. Since prostanoids were suggested to be involved in the previous animal study (Koetzler et al., 2006) we aimed to test the involvement of prostaglandin E_2_, a profound relaxant of airway smooth muscle. Following cellular activation, arachidonic acid is released from membrane PLs by phospholipase A_2_ (PLA_2_) and converted to the prostaglandin intermediate PGH_2_ either by COX‐1 (constitutively expressed in airway tissues) and COX‐2, which expression is induced by various inflammatory stimuli. Prostanoids are rapidly released, metabolized, and degraded after synthesis, suggesting their local action at the site of production. The typical actions of prostanoids include induction of the relaxation or contraction of various types of smooth muscle, modulation of neuronal activity by inhibiting or stimulating neurotransmitter release or sensitizing sensory fibers to noxious stimuli, roles in apoptosis, cell differentiation, regulation of the blood platelets activity and contribution to vascular homeostasis and hemostasis (Narumiya, 2007). The prostanoids can be categorized into two classes: stimulatory prostaglandins (PGD_2_, PGF_2α_, TxA_2_) that are potent bronchoconstrictors and inhibitory prostaglandins (PGE_2_, PGI_2_) that reduce bronchoconstrictor responses and attenuate the release of bronchoconstrictor mediators like acetylcholine from airway nerves (Barnes, 2002). PGE_2_ is normally present in human and guinea pig lung tissue and activates four different EP receptors (Säfholm et al., 2013; Vancheri et al., 2004). EP_2_ and EP_4_ receptors mediate the bronchodilation effect of PGE_2_ in guinea pigs and humans respectively (Säfholm et al., 2013) by signaling through a Gs‐protein to increase intracellular cAMP levels (Sheller et al., 2000). PGE_2_ is produced by airway smooth muscle, tracheal epithelial cells, alveolar macrophages, and pulmonary endothelial cells (Hempel et al., 1994; Meyrick et al., 1989; Pang & Knox, 1997; Widdicombe et al., 1989) eliciting anti‐inflammatory but also inflammatory effects (Kountz et al., 2021; Säfholm et al., 2013). PGE_2_ has effects on airway smooth muscle and nerves, which allow it to modulate airway caliber (Bärnthaler et al., 2017; Kountz et al., 2021). Since relaxant effect of surfactant has been found inhibited in rat epithelium denuded tissues (Koetzler et al., 2006), epithelium may be an important source of molecules released towards airway smooth muscle (Bärnthaler et al., 2017; Fujii et al., 2016; Schmidt et al., 2011) to cause relaxation. It has been reported that pulmonary surfactant and phospholipases may stimulate arachidonic acid pathway and prostaglandins production by human amnion cells (Bernal et al., 1988; López Bernal & Phizackerley, 2000). Another study (Ohtsuka et al., 1990) showed that major component of surfactant (dipalmitoyl‐phosphatidylcholine) stimulated PGE_2_ production from human amniotic membrane. Contrary, PGE_2_ stimulates surfactant production at the bronchiolar and alveolar surface (Schmitz et al., 2007; Torday et al., 1996) indicating PGE_2_ may also help to restore the surfactant layer. The human airway epithelial cells lining the conducting airways of the lung produce PGE_2_ through an enzymatic cascade tied to the activities of phospholipase A2, COX‐1 and COX‐2 enzymes, and a terminal PGE synthase. PGE_2_ stimulates airway dilation and protects against bronchoconstriction in diseases like bronchial asthma. PGE_2_ also inhibits various inflammatory processes, including histamine release, T cell migration, and cytokine production, while promoting anti‐inflammatory IL‐10 synthesis and wound healing. Additionally, PGE_2_ drives neutrophil reverse migration to dissipate inflammation. In the lung, PGE_2_ primarily exhibits bronchoprotective and anti‐inflammatory effects, making it crucial for resolving inflammation and preventing diseases like COPD and ARDS (Kountz et al., 2021). These findings led us to consider and test whether surfactant stimulates human epithelial cells to produce PGE_2_. Therefore, we incubated the human primary bronchial epithelial cells with pulmonary surfactant and measured the concentration of PGE_2_ in cell culture samples. We proved, that PGE_2_ was significantly elevated in cell culture medium of bronchial epithelial cells exposed to pulmonary surfactant. We further tested prostanoid synthesis and EP_2_‐receptor involvement in relaxant effect of surfactant in guinea pig tissue. Endogenous PGE_2_, synthesized predominantly by COX‐2 pathway, maintains the spontaneous tone of guinea pig trachea by a balance between contractile EP_1_ receptors and relaxant EP_2_ receptors (Säfholm et al., 2013). Indomethacin is a non‐selective blocker of prostanoid synthesis and is used in tissue organ bath experiments to inhibit spontaneous tone of guinea pig ASM. In the study on rats (Koetzler et al., 2006), indomethacin inhibited relaxant effect of surfactant on precontracted bronchial tissues. In our study on guinea pigs, surfactant incubated with tracheal and lung tissue strips reduced tension of airway smooth muscle. The relaxant effect of surfactant on lung tissue was diminished when prostanoid synthesis was blocked by non‐selective COX inhibitor indomethacin and/or after EP_2_‐receptor antagonism with PF‐04418948. Surfactant reduced tension of tracheal tissues in indomethacin presence and EP_2_‐receptor antagonism; however, this effect was reduced in blockers presence. Relaxation of ASM after PGE_2_ is mediated by two receptors EP_2_ and EP_4_, both detected in guinea pigs and human. In guinea pigs we antagonized EP_2_ receptor and in human ASM cells EP_4_ receptor due to their dominant expression reported (Säfholm et al., 2013). PGE_2_ mediates relaxation of guinea pig airway smooth muscle predominantly through EP_2_ receptors and epithelial COX‐2 pathway (Säfholm et al., 2013), while relaxant EP_4_ receptors and COX‐1 have a significant role in PGE_2_ mediated relaxation in human airways (Buckley et al., 2011; Harrington et al., 2008; Joshi et al., 2021; Peebles, 2019). Theoretically, relaxation could occur through both EP_2_ and EP_4_ receptors, therefore, the involvement of both receptors needs to be further tested. Since, surfactant still relaxed the tracheal tissue in EP_2_‐receptor antagonism and/or prostanoid synthesis inhibition, even though the relaxant effect was reduced, it may indicate other mechanisms to be involved and EP receptors may be partially mediating the relaxant effect of surfactant. Some of them have been already ruled out in other studies (Calkovska et al., 2015; Koetzler et al., 2006). Distinct components of surfactant as unsaturated phosphatidylcholine, disaturated dipalmitoyl‐phosphatidylcholine, phosphatidylglycerol and surfactant protein A have been tested separately in tissue organ baths and it was revealed they relax rat precontracted bronchial tissue (Koetzler et al., 2006). Surfactant is a complex molecule composed of several PLs, neutral lipids, and proteins covering the airway epithelium composed of different cell types (Hasleton et al., 2012) potentially interacting with pulmonary surfactant. The disturbed surfactant layer in asthmatics with hyperreactive airways (Hohlfeld et al., 1999) or surfactant's property to mask the irritant epithelial receptors have been reported (Hills, 1996; Hills & Chen, 2000) suggesting protective barrier effect of pulmonary surfactant as another potential mechanism of how surfactant may promote ASM relaxation. The neuro‐epithelial cell bodies (NEB) and smooth muscle‐associated airway receptors (SMARs) found in the proximity of club cells producing surfactant proteins in terminal bronchioles are airway intraepithelial receptors regulating airway smooth muscle tone (Adriaensen et al., 1985) and their interaction in regard to surfactant relaxant effect needs to be further studied.

So far, the direct relaxant effect of surfactant on airway smooth muscle has been described only by the tissue organ bath method on rodents (Calkovska et al., 2015; Koetzler et al., 2006; Topercerova et al., 2019). This study introduces an alternative method to the tissue organ bath approach, using human airway smooth muscle cell cultures and cell stiffness measurements to investigate the relaxant effect of pulmonary surfactant. This is consistent with other studies measuring ASM cell stiffness after contractile agonists methacholine, histamine, carbachol, serotonin, or bradykinin which led to cell stiffening (An et al., 2012, 2016) or relaxant agonists isoprotenerol and cell‐permeable cAMP analog (db‐cAMP) which led to cell softening (An et al., 2012; Deshpande et al., 2010). Contraction of the ASM cell is the result of intracellular calcium concentration increase and myosin light chain kinase (MLCK) phosphorylation of the light chain of myosin, and, in conjunction with actin, cross‐bridge cycling occurs, initiating shortening of the smooth muscle cell. Relaxation of the ASM is the result of decreased intracellular calcium concentration, increased MLC phosphatase activity to dephosphorylate the light chain of myosin, and cross‐bridge detachment (Webb, 2003). The study demonstrates that cell stiffness measurements accurately reveal the contractile state of airway smooth muscle cells, reproducing results from the organ bath method. The measurement requires only one single cell to be contacted by the AFM cantilever and the stiffness data can be measured instantly upon the contact. Mapping of airway smooth muscle cell stiffness can help to observe contractile/relaxant events by activating/deactivating of potential receptors involved. Prostanoids were indicated to be involved in surfactant relaxant effect (Koetzler et al., 2006); therefore, we decided to test whether surfactant decrease the stiffness through EP_4_ receptors of the profound ASM relaxant, prostaglandin E_2_. Both, PGE_2_ and pulmonary surfactant used in our experiments led to decrease in the ASM cell stiffness; however, ASM cell stiffness increased after surfactant if EP_4_ receptors for PGE_2_ were antagonized. We report that blockade of PGE_2_ membrane receptors prevents decrease in stiffness and works antagonistically to pulmonary surfactant's relaxant effect. This observation suggests that PGE_2_‐related receptor EP_4_ may be involved in surfactant softening of the ASM cells. However, the study's limitation is that it does not account for the role of airway epithelial cells in mediating the relaxant effect of surfactant. Future AFM experiments with co‐cultures of airway epithelial cells and airway smooth muscle cells are required to address this limitation. Since human ASM cells contain phospholipases in plasma membrane or cytosol (Mizuta et al., 2011; Pascual et al., 2003; Rhee & Bae, 1997; Sayers et al., 2006) hydrolysis of surfactant PLs might serve as an arachidonate source for PGE_2_ production by airway smooth muscle cells.

Relaxant activity of surfactant was further tested on hyperresponsive airways after ovalbumin challenge. Ovalbumin is used to induce chronic allergic inflammation of airways (Ohki et al., 2002) and bronchial hyperresponsiveness (Warren et al., 2019). Ovalbumin challenge lasting 21 days increased sensitivity and tension of tracheal and lung tissue strips. Ongoing allergic inflammation was further proved by increased eosinophils in bronchoalveolar lavage fluid, elevated concentration of IL‐5 in lung tissue homogenates and increased ratio between wet and dry lungs for assessment of lung oedema formation (Figures [Link], [Link]). The accumulation of eosinophils and formation of lung oedema is a common feature of allergic airway inflammation and correlates with disease severity. Lung oedema contributes to mucous swelling resulting in airway narrowing (Snashall & Chung, 2012) and bronchospasm (Hagberg, 2013; Jo et al., 2013). Inflammation is characterized by infiltration of the airway by inflammatory cells including eosinophils, mast cells, monocytes, lymphocytes, and neutrophils, which contribute to elevated levels of inflammatory mediators and pro‐contractile molecules (Khan, 2013). IL‐5 is involved in airway eosinophilia and contribute to the development of bronchial hyperreactivity in ovalbumin challenged guinea pigs (Kraneveld et al., 1997; Van Oosterhout et al., 2012). Surfactant in our experiments reduced tension of healthy and ovalbumin challenged trachea; however, relaxant effect was milder on ovalbumin challenged trachea. Surfactant reduced tension of healthy lung tissues; however, relaxant effect of surfactant on challenged lungs was not significant from the data collected. Pulmonary surfactant may be impaired by infection leading to disturbed alveolar‐capillary permeability, alveolar type 2 cell damage or inflammatory reaction, all leading to surfactant deficiency or inhibition (Herting & Robertson, 2005). In conditions like bronchial asthma characteristic with allergic inflammation of airways, it has been demonstrated that there is a surfactant dysfunction mainly due to inhibition by proteins that enter the airways during the inflammatory process (Hohlfeld, 2002). Dysfunction of surfactant in ovalbumin‐induced asthma model more likely disturbs intra‐alveolar pool of surfactant because of the inflammatory reaction after allergen challenge and does not influence the intracellular surfactant pool. Stereological method revealed that the alveolar type II cells or the lamellar bodies storage organelles of surfactant did not exhibit any damage or significant quantitative alterations after ovalbumin challenge (Schmiedl et al., 2023). Allergic airway inflammation affects not only bronchiolar airspace but causes alveolar dysfunction contributing to the pathology of allergic asthma. House‐dust mite induced allergic inflammation leading to alveolar macrophage death, pneumocyte hypertrophy and surfactant dysfunction. Allergic lung surfactant showed reduced efficiency to form surface‐active film due to reduced protein levels of SP‐B and SP‐C (Feo‐Lucas et al., 2023). Tracheal tissue consists of extrapulmonary airway smooth muscle; however, lung tissue contains intrapulmonary vascular and bronchial airway smooth muscle mechanically interacting (Loring, 2017) with differences in phenotype, activators, and content of contractile proteins (Barnes, 2012; Halayko et al., 1996). Tension calculated from our readings informs us about the effect of surfactant on extra‐ (trachea) and intrapulmonary airways (lungs), a predominant site of inflammation and airflow obstruction in asthmatic patients (Tulic et al., 2001).

Contractile response of tracheal tissues to methacholine was reduced after surfactant. This phenomenon is suspicious for masking effect of surfactant on muscarinic M_3_ receptors, typically activated by methacholine. Masking or barrier function of surfactant has been described well by Professor Hills (Hills et al., 2003; Hills & Chen, 2000), explaining that surfactant barriers are present in different regions of human body as tendons, articular or pleural surfaces, gastric mucosa, eustachian tube to either lubricate or prevent against diffusion of potential negative ions (Mills et al., 2005). The fact is that surfactant barrier serves as a semipermeable membrane pumping water and preventing leakage of protein at the alveolar surface. Leaked serum proteins can carry away insoluble lipids of surfactant and erode the lipid barrier. A partial physical barrier separating receptors on bronchial epithelium and neuroepithelial cells from bronchial air may regulate broncho‐constrictive reflex to noxious and airborne stimuli. Surface‐active PL (SAPL) was used in rat airways, where reduced neural response to a methacholine challenge by an amount comparable to Salbutamol (Hills & Chen, 2000). Inhalation of surfactant Surfacen® relieved bronchoconstriction in murine allergen‐induced acute asthma model comparable to Salbutamol. Surfacen® inhibited Th2 inflammation by lowering levels of IL‐5 and IL‐13 and increasing INF‐γ in bronchoalveolar lavage, decrease of serum specific IgE, increase of IgG2a antibody, suggesting potential anti‐allergy effects towards Th1‐immune response (Blanco et al., 2022). On the other hand, sputum from asthmatic patients contains granulocyte‐derived lipid droplets and extracellular vesicles containing sphingolipids, arachidonic acid containing lipids as phosphatidylcholine, cholesterol and others worsening surfactant ability to reduce the surface tension and compromising its role as an immunologic barrier (Brandsma et al., 2023).

The study investigates the effect of surfactant on airway smooth muscle tone and the potential role of prostaglandin E_2_ and its receptors. In the in vivo situation, surfactant comes into contact with the airway epithelium suggesting its effect on ASM is indirect. The study has shown that surfactant increases PGE_2_ production of human bronchial epithelial cells, suggesting that PGE_2_ may act as a second messenger. The relaxant effect of a surfactant is reduced in the tracheal tissue of guinea pigs when prostanoid synthesis and EP_2_ receptors are blocked. This confirms the role of prostanoids and EP_2_ receptors in this process and implies that other mechanisms may be involved. Interestingly, surfactant directly relaxes human ASM cells in the absence of epithelium and the relaxation is inhibited if EP_4_ receptors are blocked. The affinity between surfactant and the EP_4_ receptor supports the hypothesis that PGE_2_ and/or its related EP_4_ receptor may be involved in surfactant‐epithelium‐mediated relaxation of ASM. To further investigate the interaction between surfactant and ASM, co‐cultures of epithelium and airway smooth muscle could be used. The preliminary results presented in this study provide a basis for generating hypotheses for future research on the relaxant function of pulmonary surfactant in the airways, with the potential for more extensive sampling in future studies.

## AUTHOR CONTRIBUTIONS

Hanusrichterova J, Kolomaznik M, Barosova R, Adamcakova J, Mokra D, Mokry J, and Calkovska A performed experiments on tissue organ bath system. Hanusrichterova J analyzed data of experiments on tissue organ bath system. Kelly MM and de Heuvel E designed research of airway smooth muscle cell stiffness experiments and performed immunohistochemistry staining procedure for alpha smooth muscle actin. Amrein MW and Mukherjee PG designed and supervised the single cell force spectroscopy (SCFS) using AFM. Hanusrichterova J, Skovierova H, Wiehler S, and Proud D designed research, performed experiments, analyzed data, interpreted results of experiments on primary human bronchial epithelial cells. Hanusrichterova J, Mukherjee PG, and Amrein MW performed experiments, interpreted results of atomic force microscope experiments on airway smooth muscle cells. Shen H designed and supervised the statistical analysis of the SCFS experiments on airway smooth muscle cells. Hanusrichterova J prepared figures. Hanusrichterova J, Calkovska A, Mokry J, and Amrein MW drafted manuscript, edited, and revised manuscript. Calkovska A, Mokry J, and Amrein MW approved final version of manuscript.

## FUNDING INFORMATION

The Slovak Research and Development Agency, Slovak Republic: APVV‐17‐0250 (to tissue organ bath experiments and human bronchial epithelial cell experiments). The Scientific Grant Agency, Slovak Republic: VEGA 1/0055/19 (to tissue organ bath experiments). Ministry of Education, Science, Research and Sport of the Slovak Republic; The National Scholarship Programme of the Slovak Republic for the support of mobility of students, PhD students, university teachers, researchers, and artists/26246 and 34237 (to first author Hanusrichterova J for the internship at the University of Calgary to perform experiments on atomic force microscope). The Microscopy and Imaging Facility of the Cumming School of Medicine, University of Calgary provided the AFM and cell culture facilities for this manuscript.

## CONFLICT OF INTEREST STATEMENT

The authors declare no conflicts of interest.

## Supporting information


Data S1.

Figure S1.

Figure S2.

Figure S3.

Figure S4.

Figure S5.

Figure S6.

Figure S7.

Figure S8.

## Data Availability

The data supporting the findings of this study will be openly available upon reasonable request to the corresponding author.
