# Supplementary material for: Pulmonary surfactant and prostaglandin E2 in airway smooth muscle relaxation of human and male guinea pigs
Source: Physiol Rep. 2024 Sep 8;12(17):e70026. doi: 10.14814/phy2.70026 (PMC11381196; doi:10.14814/phy2.70026)
Supplement: Supplementary file 1 — Data S1. [file PHY2-12-e70026-s001.docx]

Pulmonary surfactant and prostaglandin E_2_ in airway smooth muscle relaxation of human and male guinea pigs

Hanusrichterova J^1^, Kolomaznik M^1^, Barosova R^2^, Adamcakova J^2^, Mokra D^2^, Mokry J^3^, Skovierova H^1^, Kelly MM^4^, de Heuvel E^4^, Wiehler S^5^, Proud D^5^, Shen H^6^, Mukherjee PG^7^, Amrein MW^7^, Calkovska A^2^

**Supplemental material**

^1^Biomedical Centre Martin, Jessenius Faculty of Medicine in Martin, Comenius University in Bratislava, Martin, Slovakia

^2^Department of Physiology, Jessenius Faculty of Medicine in Martin, Comenius University in Bratislava, Martin, Slovakia

^3^Department of Pharmacology, Jessenius Faculty of Medicine in Martin, Comenius University in Bratislava, Martin, Slovakia

^4^Department of Pathology and Laboratory Medicine, Cumming School of Medicine, University of Calgary, Calgary, Alberta, Canada

^5^Department of Physiology and Pharmacology and Snyder Institute for Chronic Diseases, Cumming School of Medicine, University of Calgary, Calgary, Alberta, Canada

^6^Department of Mathematics and Statistics, Faculty of Science, University of Calgary, Alberta, Canada

^7^Department of Cell Biology and Anatomy, Cumming School of Medicine, University of Calgary, Calgary, Alberta, Canada

**Reagents and any unique materials used**

**Supplemental table S1.** Tissue organ bath experiments

| Material | Company name | Catalog # |
| --- | --- | --- |
| Exogenous pulmonary surfactant CUROSURF® (poractant alfa) | Chiesi Pharmaceutici, Parma, Italy | 10122-510-03 |
| Methacholine chloride | Merck KGaA, Darmstadt, Germany | PHR1943 |
| Indomethacin | Merck KGaA, Darmstadt, Germany | I7378-5G |
| Albumin from chicken egg white | Merck KGaA, Darmstadt, Germany | A5503-10G |
| Dimethyl sulfoxide | Merck KGaA, Darmstadt, Germany | D8418-100ML |
| ELISA Kit for Interleukin 5 (IL5) | Cloud-Clone Corporation, Houston, Texas, USA | SEA078Hu |
| PF-04418948 | Cayman Chemicals, Ann Arbor, Michigan, USA | PZ0213-5MG |
| Ultrapure water for PGE2 Elisa kit | Cayman Chemicals, Ann Arbor, Michigan, USA | 400000 |
| Aluminum hydroxide | Centralchem s.r.o., Bratislava, Slovakia | L00564-500G |

**Supplemental table S2.** Airway smooth muscle cells staining for alpha smooth muscle actin

| Material | Company name | Catalog # |
| --- | --- | --- |
| Primary antibody Mouse Alpha-Smooth Muscle Actin Monoclonal Antibody (1A4 (asm-1)), Biotin; 1:100 diluted | Thermofisher Scientific Inc., Mississauga, Ontario, Canada | MA5-11544 |
| Secondary antibody Goat Anti-Mouse IgG Antibody (H+L), Biotinylated; 1:200 diluted | Vector Laboratories, Inc..; Newark, California, USA | BA-9200-1.5 |

**Supplemental table S3.** Atomic force microscope experiments

| Material | Company name | Catalog # |
| --- | --- | --- |
| Cantilever HYDRA6R-200N-SiO2-A-GG-5; 5-9 μm SiO_2_ spheres coated with gold attached to HYDRA6R-200NGG-TL | AppNano, Inc., Mountain View, California, USA | Custom made |
| Bovine lipid extract surfactant BLES | BLES Biochemicals Inc., London, ON, Canada | 3004 90.90A |
| DMEM (Dulbecco's Modified Eagle Medium), high glucose, pyruvate | Thermofisher Scientific Inc., Mississauga, Ontario, Canada, Gibco™ | 11995065 |
| Trypsin-EDTA (0.25%), phenol red | Thermofisher Scientific Inc., Mississauga, Ontario, Canada, Gibco™ | 2520072 |
| Amphotericin B 250 μg/mL 50ML | Thermofisher Scientific Inc., Mississauga, Ontario, Canada, Gibco™ | 15290026 |
| Hanks' Balanced Salt Solution (HBSS), no calcium, no magnesium, no phenol red | Thermofisher Scientific Inc., Mississauga, Ontario, Canada, Gibco™ | 14175095 |
| Penicillin-Streptomycin (10,000 U/mL) | Thermofisher Scientific Inc., Mississauga, Ontario, Canada, Gibco™ | 15140122 |
| Fetal bovine serum (FBS) qualified Canada origin 10x50mL | Thermofisher Scientific Inc., Mississauga, Ontario, Canada, Gibco™ | A3160702 |
| Bovine serum albumin | MilliporeSigma Canada Co., Oakville, ON, Canada | A9418-10G |
| Methacholine chloride | MilliporeSigma Canada Co., Oakville, ON, Canada | PHR1943 |
| Prostaglandin E2 (PGE_2_) | MilliporeSigma Canada Co., Oakville, ON, Canada | P0409-1MG |
| ONO-AE3-208 | MilliporeSigma Canada Co., Oakville, ON, Canada | SML2076-5MG |
| Cell culture flask, T-75 | Corning Inc., NY, USA | 430641 |
| µ-Dish 35 mm, low Grid-500 | ibidi GmbH, Gräfelfing, Germany | 80156 |

**Supplemental table S4.** Epithelial cell experiments

| Material | Company name | Catalog # |
| --- | --- | --- |
| Bovine lipid extract surfactant BLES | BLES Biochemicals Inc., London, ON, Canada | 3004 90.90A |
| Bronchial Epithelial Cells with retinoic acid | Lonza Walkersville, Maryland, USA | CC2540 |
| Trypsin/EDTA 100 mL | Lonza, Walkersville, Maryland, USA | CC-5012 |
| Trypsin Neutralizing Solution 100 mL | Lonza, Walkersville, Maryland, USA | CC-5002 |
| HEPES Buffered Saline Solution 100 mL | Lonza, Walkersville, Maryland, USA | CC-5022 |
| BEBM^TM^ Basal Medium | Lonza, Walkersville, Maryland, USA | CC-3171 |
| BEGM^TM^ SingleQuotsTM Supplement Pack | Lonza, Walkersville, Maryland, USA | CC-4175 |
| Cell culture flask, T-75 | Sarstedt, Nümbrecht, Germany | 83.3911.302 |
| Cell culture plate, 6 well | Sarstedt, Nümbrecht, Germany | 83.3920.300 |
| Prostaglandin E2 ELISA Kit Monoclonal | Cayman Chemicals, Ann Arbor, Michigan, USA | 514010 |

**Ovalbumin challenge of guinea pigs (markers of allergic inflammation)**

White blood cell differential analysis

The total white blood cell counts, and individual subpopulations were evaluated in the samples of blood and bronchoalveolar lavage fluid (BALF) by an automatic hematology analyzer (Sysmex XT-2000i, Landskrona, Sweden) immediately upon collection and read in absolute values (cell count×10^9^/L). Blood samples were collected after the heart puncture (arterial blood from left ventricle) and samples of BALF were recovered from left lung lobes after double lavage with the saline solution heated to 37 °C at the dose of 10 mL/kg b.w..

Ovalbumin challenge of animals led to an increase in number of eosinophils in bronchoalveolar lavage fluid (BALF) (Supplemental Figure S1).


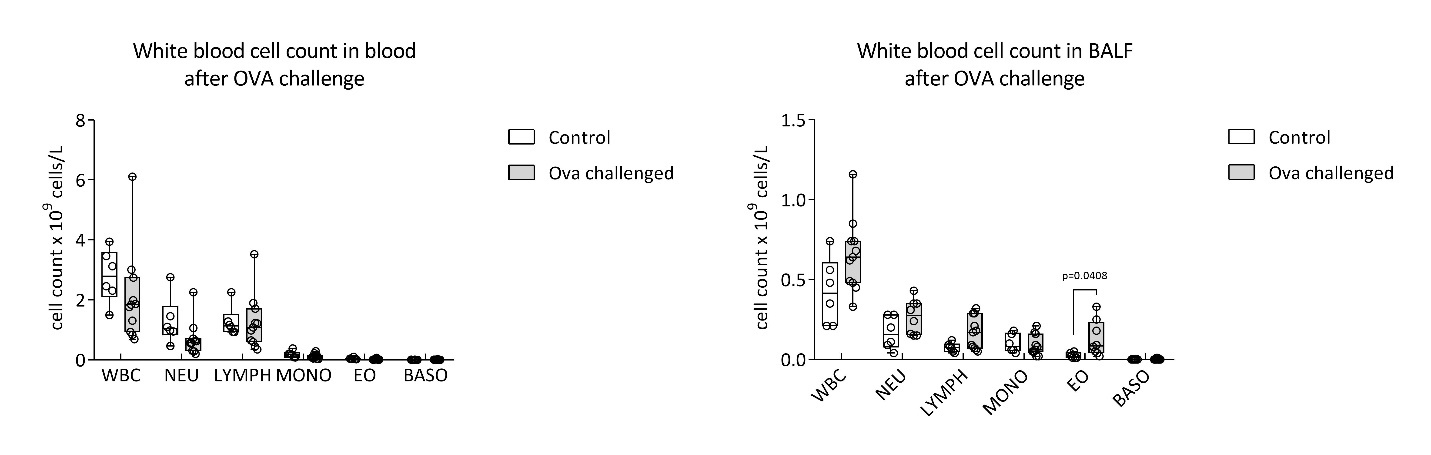


**Supplemental Figure S1.** Total white blood cell (WBC) count and differential WBC count; neutrophils (NEU), lymphocytes (LYMPH), monocytes (MONO), eosinophils (EO), basophils (BASO) (number of cells × 10^9^/L) in blood and bronchoalveolar lavage fluid (BALF) of animals after 21-day ovalbumin challenge (Ova challenged). Control n=6; Ova challenged n=6-11. All data points (min to max) are displayed with median line. Comparisons made by two-tailed unpaired Student’s t-test.

**Lung tissue homogenates**

Right lung tissue pieces were homogenized in ice-cold PBS (0.02 mol/L at pH 7.2) for final concentration 10 % (weight/volume). Homogenized samples were sonicated for 30 seconds, underwent two freeze-thaw cycles, and were centrifuged for 15 min at 4 °C and 1,500 x g. Supernatants were used for further analysis.

Sandwich enzyme linked immunosorbent assay (ELISA) for *in vitro* quantitative measurement of interleukin 5

Lung tissue homogenates were assayed for interleukin 5 (IL-5), marker of ovalbumin-induced allergic airway inflammation associated with hyperresponsiveness of the airways. ELISA assay was performed according to the instruction manual included in the kit (Cloud-Clone Corporation, Houston, Texas, USA, cat. no.: SEA078Hu).

Interleukin 5

Concentration of interleukin 5 measured from lung tissue homogenates has increased after ovalbumin challenge compared to Control group (Supplemental Figure S2).


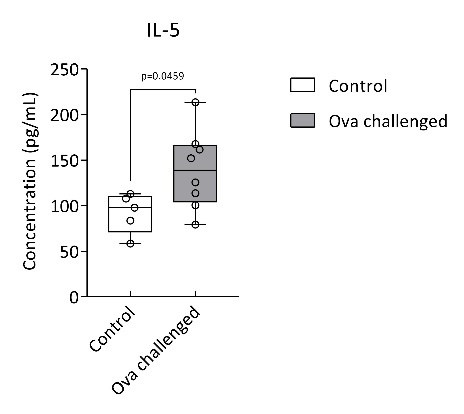


**Supplemental Figure S2.** Interleukin 5 in lung tissue homogenates of Control and ovalbumin challenged animals (Ova challenged) after 21-day ovalbumin challenge. Control n=5 animals; Ova challenged n=8. All data points (min to max) are displayed with median line. Comparison made by two-tailed unpaired Student’s t-test.

Lung oedema formation

The strips from the right middle lobe of the non-lavaged lungs were excised and used to assess lung oedema formation after ovalbumin challenge. The lungs were weighed before and after drying at room temperature for 48 hours period. The total lung oedema formation was expressed as wet-dry lung weight ratio (W/D) and compared to Control group.

Formation of lung oedema after ovalbumin challenge

The ratio between wet and dry lungs was further analyzed to confirm model of bronchial hyperresponsiveness after ovalbumin challenge. Pulmonary oedema formation was increased in ovalbumin challenged lungs (Supplemental Figure S3).


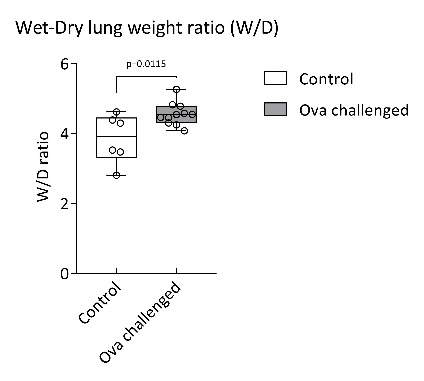


**Supplemental Figure S3.** Wet-Dry ratio comparison of Control and ovalbumin challenged animals (Ova challenged) after 21-day challenge. Control n=6; Ova challenged n=11. All data points (min to max) are displayed with median line. Comparison made by two-tailed unpaired Student’s t-test.

**Descriptive statistics of AFM results**

**Methacholine chloride**

**Supplemental Table S5.** Descriptive statistics of cell nuclei measured by AFM for Young’s Modulus evaluation at baseline (Base) and after methacholine (Mtch). Standard deviation (SD), standard error of mean (SEM), mean, and median are expressed in Pascals (Pa). Count represents number of force-curves recorded.

| Cells | Cell 1 | | Cell 2 | | Cell 3 | | Cell 4 | | Cell 5 | | Cell 6 | | Cell 7 | |
| --- | --- | --- | --- | --- | --- | --- | --- | --- | --- | --- | --- | --- | --- | --- |
| Treatment | Base | Mtch | Base | Mtch | Base | Mtch | Base | Mtch | Base | Mtch | Base | Mtch | Base | Mtch |
| Count | 64 | 64 | 64 | 64 | 64 | 64 | 64 | 64 | 59 | 63 | 144 | 141 | 160 | 160 |
| SD | 1446 | 1037 | 293.3 | 1429 | 567.7 | 680.6 | 183.5 | 778 | 155.8 | 549.5 | 445.3 | 428.2 | 207.1 | 282.4 |
| SEM | 180.8 | 129.6 | 36.66 | 178.7 | 70.96 | 85.07 | 22.93 | 97.25 | 20.28 | 69.23 | 37.10 | 36.06 | 16.37 | 22.33 |
| Mean | 3338 | 4513 | 997.4 | 3114 | 1145 | 1588 | 639.1 | 1959 | 540.5 | 1121 | 1160 | 1072 | 555.5 | 609.7 |
| Median | 3131 | 4490 | 979.8 | 3244 | 1130 | 1618 | 611.3 | 2070 | 511.9 | 919.5 | 1108 | 981.5 | 509.6 | 529.4 |

**Supplemental Table S6.** Descriptive statistics of cell cytoplasms measured by AFM for Young’s Modulus evaluation at baseline (Base) and after methacholine (Mtch). Standard deviation (SD), standard error of mean (SEM), mean, and median are expressed in Pascals (Pa). Count represents number of force-curves recorded.

| Cells | Cell 1 | | Cell 2 | | Cell 3 | | Cell 4 | | Cell 5 | | Cell 6 | | Cell 7 | |
| --- | --- | --- | --- | --- | --- | --- | --- | --- | --- | --- | --- | --- | --- | --- |
| Treatment | Base | Mtch | Base | Mtch | Base | Mtch | Base | Mtch | Base | Mtch | Base | Mtch | Base | Mtch |
| Count | 64 | 63 | 64 | 64 | 64 | 64 | 56 | 64 | 64 | 63 | 128 | 126 | 96 | 95 |
| SD | 647.2 | 1941 | 650.1 | 786.8 | 710.3 | 1261 | 502.5 | 517.2 | 456.8 | 367.5 | 496.8 | 715.6 | 200.6 | 608.1 |
| SEM | 80.90 | 244.6 | 81.26 | 98.35 | 88.79 | 157.6 | 67.15 | 64.66 | 57.10 | 46.3 | 43.91 | 63.75 | 20.47 | 62.39 |
| Mean | 2405 | 4677 | 1640 | 2974 | 2173 | 3026 | 1278 | 1483 | 951.9 | 1360 | 1484 | 1627 | 583.7 | 899.2 |
| Median | 2438 | 4073 | 1482 | 2742 | 2245 | 3075 | 1185 | 1610 | 819 | 1289 | 1453 | 1473 | 563.4 | 729.7 |

**Height and Youngs Modulus maps of ASM cells after methacholine**

Each cell was captured by light optical microscope before (baseline) and after methacholine. Next to the light microscope images, the height (µm; nm) and Young’s Modulus (kPa) force maps of analyzed cell regions are displayed. Baseline and methacholine force maps are taken from the corresponding position (we aimed to record the same region but typically encountered a slight shift during medium exchange) (Supplemental Figure S4).


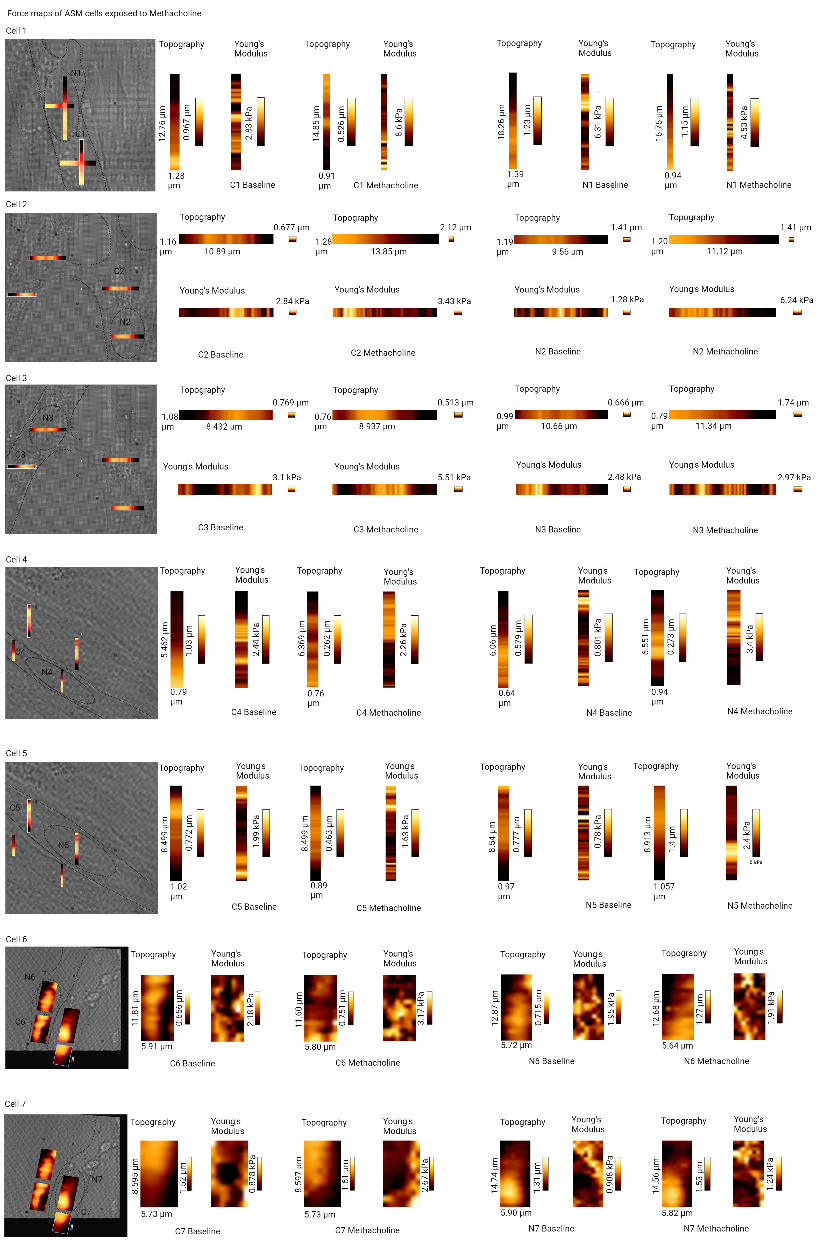


**Supplemental Figure S4.** Force maps of airway smooth muscle cells exposed to methacholine 10^-4^ M (kPa) collected by atomic force microscope. Light microscope images of the cells (63x objective) are displayed in grey with nucleus and cytoplasm denoting lines. Analyzed cell regions are displayed in red-gold force maps representing the height and Young’s Modulus of the cell regions before (baseline) and after methacholine. Cell 1 was measured in vertical and horizontal direction; vertical sections were randomly chosen for statistic evaluation.

**Descriptive statistics of AFM results**

**Prostaglandin E_2_**

**Supplemental Table S7.** Descriptive statistics of cell nuclei measured by AFM for Young’s Modulus evaluation at baseline (Base) and after PGE_2_. Standard deviation (SD), standard error of mean (SEM), mean, and median are expressed in Pascals (Pa). Count represents number of force-curves recorded.

| Cells | Cell 2 | | Cell 3 | |
| --- | --- | --- | --- | --- |
| Treatment | Base | PGE_2_ | Base | PGE_2_ |
| Count | 64 | 60 | 64 | 61 |
| SD | 608.1 | 86.40 | 188.3 | 86.86 |
| SEM | 76.01 | 11.15 | 23.53 | 11.12 |
| Mean | 1254 | 255.7 | 465 | 311.5 |
| Median | 1070 | 290 | 390.5 | 336.3 |

**Supplemental Table S8.** Descriptive statistics of cell cytoplasms measured by AFM for Young’s Modulus evaluation at baseline (Base) and after PGE_2_. Standard deviation (SD), standard error of mean (SEM), mean, and median are expressed in Pascals (Pa). Count represents number of force-curves recorded.

| Cells | Cell 1 | | Cell 2 | | Cell 3 | |
| --- | --- | --- | --- | --- | --- | --- |
| Treatment | Base | PGE_2_ | Base | PGE_2_ | Base | PGE_2_ |
| Count | 64 | 64 | 64 | 64 | 64 | 61 |
| SD | 749.8 | 62.24 | 459.5 | 85.48 | 208.6 | 96.73 |
| SEM | 93.72 | 7.781 | 57.43 | 10.69 | 26.08 | 12.38 |
| Mean | 2107 | 228.4 | 1216 | 280.2 | 909.6 | 238.5 |
| Median | 1957 | 229.4 | 1330 | 309.9 | 961.8 | 220 |

**Height and Youngs Modulus maps of ASM cells after prostaglandin E_2_**

Each cell was captured by light optical microscope before (baseline) and after PGE_2_. Next to the light microscope images, the height (µm; nm) and Young’s Modulus (kPa) force maps of analyzed cell regions are displayed. Baseline and PGE_2_ force maps are taken from the corresponding position (we aimed to record the same region but typically encountered a slight shift during medium exchange) (Supplemental Figure S5).


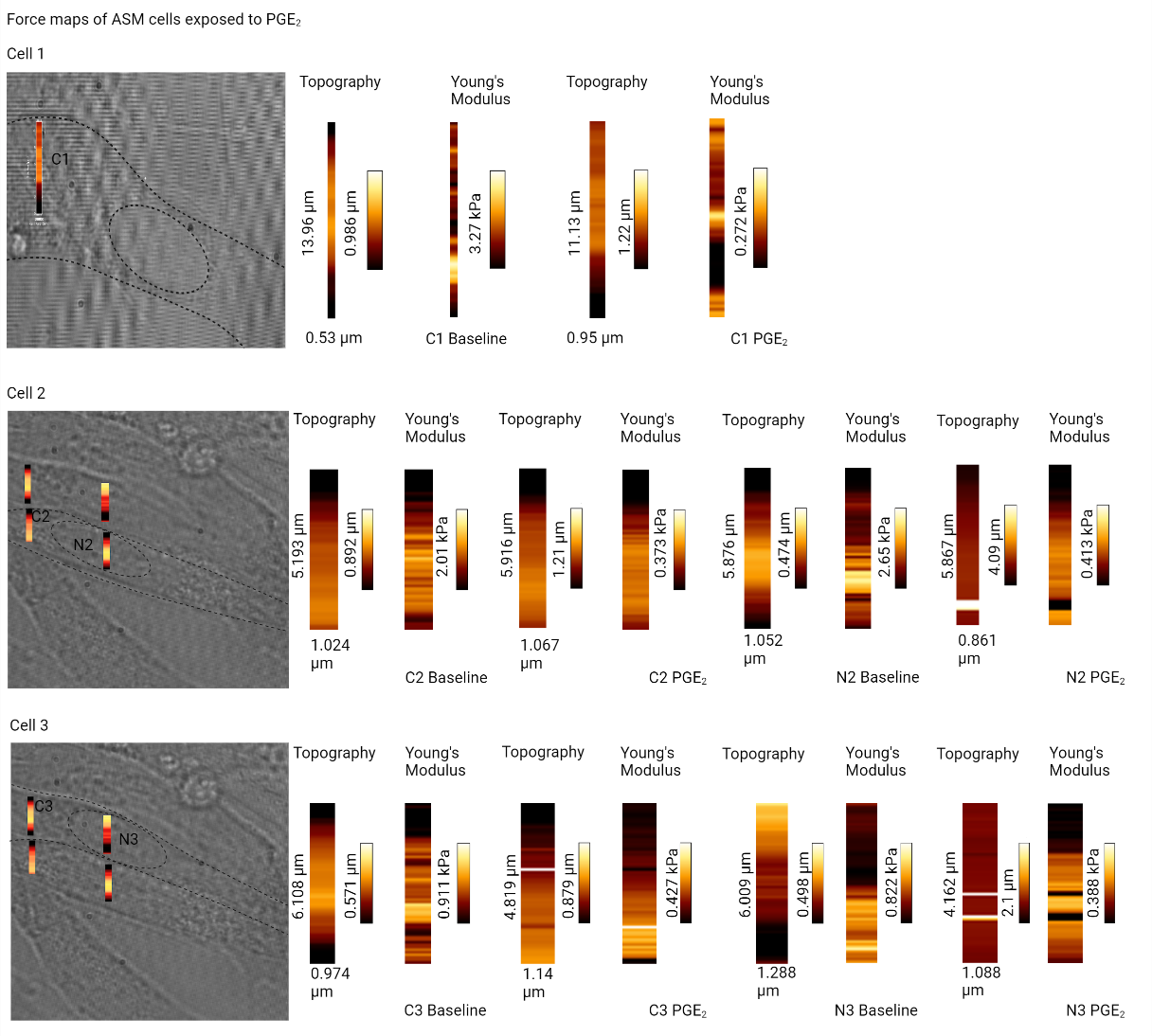


**Supplemental Figure S5.** Force maps of airway smooth muscle cells exposed to PGE_2_ (kPa) collected by atomic force microscope. Light microscope images of the cells (63x objective) are displayed in grey with nucleus and cytoplasm denoting lines. Analyzed cell regions are displayed in red-gold force maps representing the height and Young’s Modulus of the cell regions before (baseline) and after PGE_2_.

**Descriptive statistics of AFM results**

**Exogenous pulmonary surfactant BLES before and after EP_4_ receptor** **antagonism**

**Supplemental Table S9.** Descriptive statistics of cell nuclei measured by AFM for Young’s Modulus evaluation at baseline (Base) and after BLES surfactant before and after EP_4_ receptor antagonism with ONO-AE3-208 (Base_ONO_ and BLES_ONO_). Standard deviation (SD), standard error of mean (SEM), mean, and median are expressed in units of Pascals (Pa). Count represents number of force-curves recorded.

| Cells | Cell 1 | | Cell 2 | | Cell 3 | | Cell 4 | |
| --- | --- | --- | --- | --- | --- | --- | --- | --- |
| Treatment | Base | BLES | Base | BLES | Base | BLES | Base | BLES |
| Count | 144 | 160 | 160 | 223 | 184 | 160 | 216 | 198 |
| SD | 445.3 | 323 | 207.1 | 206.7 | 639.1 | 543.5 | 1066 | 1185 |
| SEM | 37.1 | 25.54 | 16.37 | 13.84 | 47.11 | 42.96 | 72.55 | 84.24 |
| Mean | 1160 | 917.7 | 555.5 | 500 | 1080 | 994.4 | 2896 | 2681 |
| Median | 1108 | 867 | 509.6 | 454.9 | 875.1 | 867.4 | 2915 | 2444 |
| After EP_4_ receptor antagonism with ONO-AE3-208 | | | | | | | | |
| Treatment | Base_ONO_ | BLES_ONO_ | Base_ONO_ | BLES_ONO_ | Base_ONO_ | BLES_ONO_ | Base_ONO_ | BLES_ONO_ |
| Count | 128 | 152 | 135 | 172 | 224 | 152 | 200 | 208 |
| SD | 598.5 | 598.3 | 670.5 | 918.2 | 739.4 | 839 | 755.4 | 945.2 |
| SEM | 52.9 | 48.53 | 57.71 | 70.01 | 49.4 | 68.05 | 53.41 | 65.53 |
| Mean | 1384 | 1471 | 1439 | 1488 | 1780 | 2267 | 1909 | 2487 |
| Median | 1264 | 1424 | 1417 | 1273 | 1653 | 2309 | 1665 | 2285 |

**Supplemental Table S10.** Descriptive statistics of cell cytoplasms measured by AFM for Young’s Modulus evaluation at baseline (Base) and after BLES surfactant before and after EP_4_ receptor antagonism with ONO-AE3-208 (Base_ONO_ and BLES_ONO_). Standard deviation (SD), standard error of mean (SEM), mean, and median are expressed in units of Pascals (Pa). Count represents number of force-curves recorded.

| Cells | Cell 1 | | Cell 2 | | Cell 3 | | Cell 4 | | Cell 5 | |
| --- | --- | --- | --- | --- | --- | --- | --- | --- | --- | --- |
| Treatment | Base | BLES | Base | BLES | Base | BLES | Base | BLES | Base | BLES |
| Count | 128 | 152 | 96 | 200 | 240 | 240 | 176 | 157 | 96 | 110 |
| SD | 496.8 | 541.4 | 200.6 | 402.4 | 655.5 | 726.1 | 1017 | 1203 | 331.4 | 213.9 |
| SEM | 43.91 | 43.91 | 20.47 | 28.46 | 42.31 | 46.87 | 76.62 | 95.97 | 33.83 | 20.39 |
| Mean | 1484 | 1374 | 583.7 | 1058 | 1502 | 1822 | 2707 | 2648 | 1479 | 527.1 |
| Median | 1453 | 1357 | 563.4 | 1031 | 1385 | 1683 | 2569 | 2432 | 1428 | 471 |
| After EP_4_ receptor antagonism with ONO-AE3-208 | | | | | | | | |  |  |
| Treatment | Base_ONO_ | BLES_ONO_ | Base_ONO_ | BLES_ONO_ | Base_ONO_ | BLES_ONO_ | Base_ONO_ | BLES_ONO_ |  |  |
| Count | 120 | 96 | 184 | 144 | 224 | 155 | 207 | 116 |  |  |
| SD | 676.3 | 876.6 | 408.5 | 823 | 492.9 | 1157 | 467.5 | 699.2 |  |  |
| SEM | 61.74 | 89.47 | 30.11 | 68.59 | 32.93 | 92.92 | 32.49 | 64.92 |  |  |
| Mean | 1807 | 2082 | 1561 | 2109 | 1457 | 1435 | 1721 | 2389 |  |  |
| Median | 1715 | 1899 | 1612 | 2093 | 1418 | 999.3 | 1750 | 2476 |  |  |

**Height and Youngs Modulus maps of ASM cells after exogenous pulmonary surfactant BLES before and after EP_4_ receptor antagonism**

Each cell was captured by light optical microscope before (baseline) and after BLES surfactant and this was repeated in the presence of EP_4_ receptor antagonist ONO-AE3-208. Next to the light microscope images, the height (µm; nm) and Young’s Modulus (kPa) force maps of analyzed cell regions are displayed. Baseline and BLES force maps are taken from the corresponding position (we aimed to record the same region but typically encountered a slight shift during medium exchange) (Supplemental Figure S6).


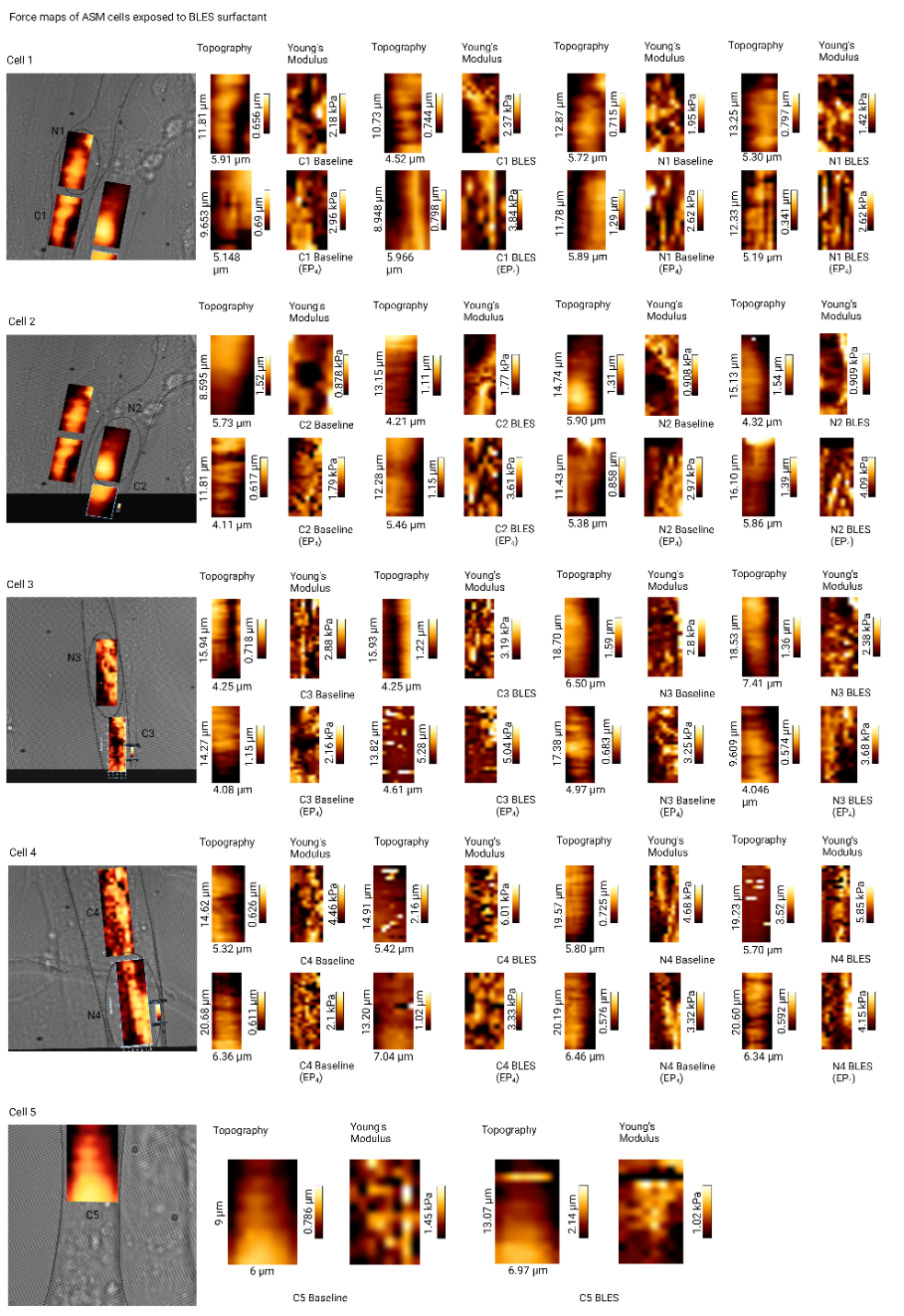


**Supplemental Figure S6.** Force maps of airway smooth muscle cells exposed to BLES surfactant after EP_4_ receptor antagonism (kPa) collected by atomic force microscope. Light microscope images of the cells (63x objective) are displayed in grey with nucleus and cytoplasm denoting lines. Analyzed cell regions are displayed in red-gold force maps representing the height and Young’s Modulus of the cell regions before (baseline) and after BLES surfactant.

**AFM results**

**The effect of sterile water on airway smooth muscle cell stiffness**

Incubation of airway smooth muscle cells with sterile water did not result in a significant difference in the cell stiffness before (baseline) and after treatment (sterile water) of both the nucleus (p = 1.0000) and cytoplasm (p = 1.000000), as illustrated in Supplemental Figure S7. The linear mixed model was applied to analyze repeated measurements of nucleus and cytoplasm values at baseline and after sterile water across 4 cells, considering both fixed and random effects. In simple terms, the treatment (sterile water) does not appear to have any effect on the outcome based on this model. However, there is a significant decrease in the response value of both nucleus and cytoplasm when using the surfactant treatment compared to the non-surfactant treatment (p-value < 2.2e-16; not shown in the figure).

**
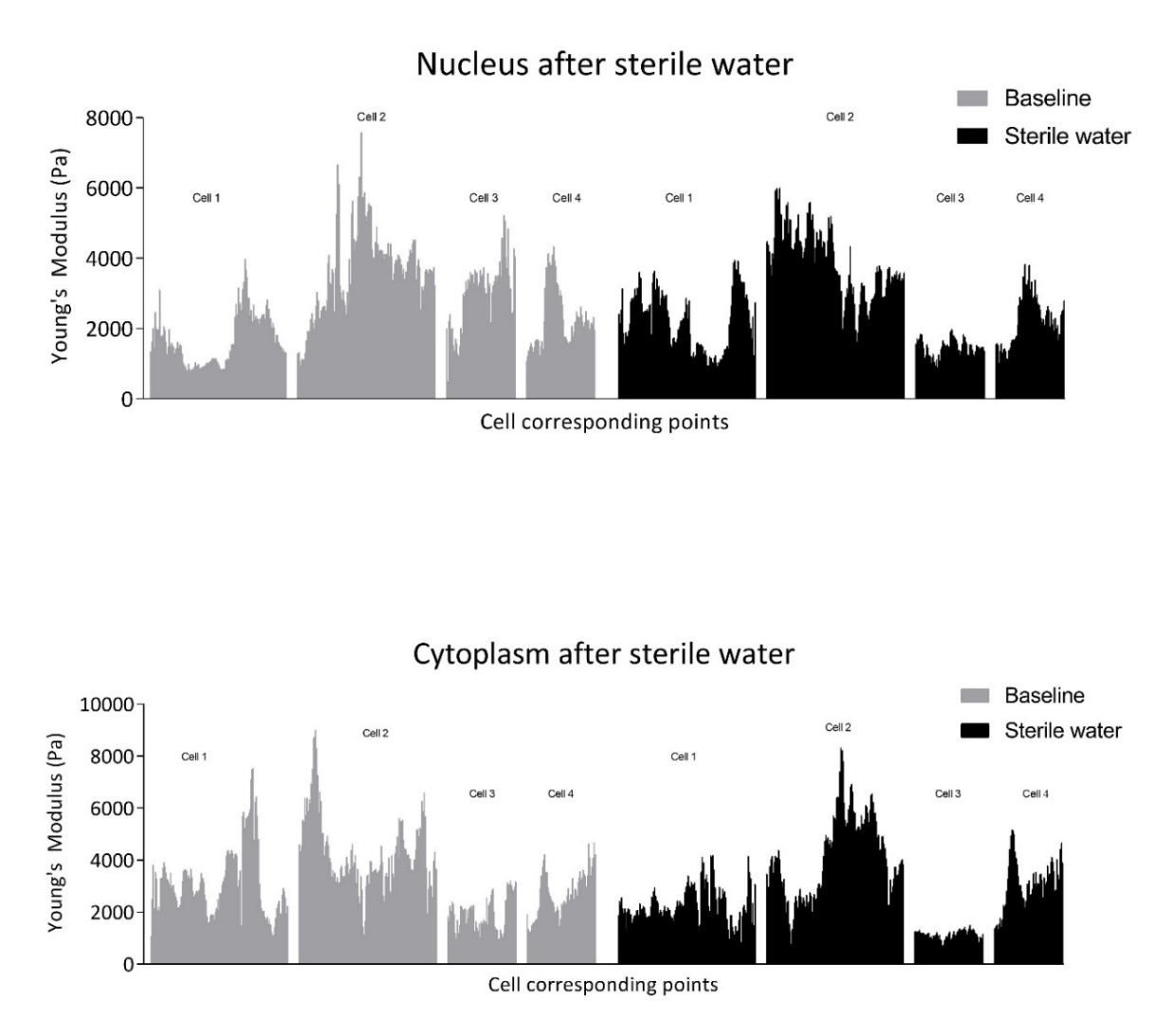
**

**Supplemental Figure S7.** Airway smooth muscle cell stiffness of nucleus and cytoplasm after sterile water. Stiffness is expressed as Young’s Modulus (Pa). Stiffness of each cell (n=4 cells) was recorded at minimum of 64 cell points at baseline and after 5-minute incubation with sterile water (0.01% content in the cell medium). Cell corresponding points measured at baseline and after sterile water were taken from the same cell, but the points are not identical. The all data points are displayed. Comparison made by Linear mixed model and the Type III Wald F test.

**Descriptive statistics of AFM results**

**Sterile water**

**Supplemental Table S11.** Descriptive statistics of cell nuclei measured by AFM for Young’s Modulus evaluation at baseline (Base) and after sterile water (Water). Standard deviation (SD), standard error of mean (SEM), mean, and median are expressed in Pascals (Pa). Count represents number of force-curves recorded.

| Cells | Cell 1 | | Cell 2 | | Cell 3 | | Cell 4 | |
| --- | --- | --- | --- | --- | --- | --- | --- | --- |
| Treatment | Base | Water | Base | Water | Base | Water | Base | Water |
| Count | 126 | 127 | 128 | 128 | 64 | 64 | 64 | 64 |
| SD | 724.7 | 832.7 | 1245 | 978.9 | 966.8 | 240.9 | 852.5 | 708.8 |
| SEM | 64.56 | 73.89 | 110 | 86.52 | 120.8 | 30.11 | 106.6 | 88.6 |
| Mean | 1682 | 2243 | 3654 | 3902 | 3061 | 1455 | 2309 | 2276 |
| Median | 1503 | 2279 | 3700 | 3752 | 3292 | 1468 | 2167 | 2269 |

**Supplemental Table S12.** Descriptive statistics of cell cytoplasms measured by AFM for Young’s Modulus evaluation at baseline (Base) and after sterile water (Water). Standard deviation (SD), standard error of mean (SEM), mean, and median are expressed in Pascals (Pa). Count represents number of force-curves recorded.

| Cells | Cell 1 | | Cell 2 | | Cell 3 | | Cell 4 | |
| --- | --- | --- | --- | --- | --- | --- | --- | --- |
| Treatment | Base | Water | Base | Water | Base | Water | Base | Water |
| Count | 128 | 128 | 128 | 128 | 64 | 64 | 64 | 64 |
| SD | 1355 | 689.1 | 1420 | 1595 | 643.9 | 160.3 | 867.8 | 939.4 |
| SEM | 119.8 | 60.91 | 125.5 | 140.9 | 80.49 | 20.04 | 108.5 | 117.4 |
| Mean | 3150 | 2317 | 4271 | 4167 | 1949 | 1144 | 2733 | 3152 |
| Median | 2944 | 2169 | 3942 | 4045 | 1944 | 1159 | 2627 | 3128 |

**Height and Youngs Modulus maps of ASM cells after sterile water**

Each cell was captured by light optical microscope before (baseline) and after sterile water. Next to the light microscope images, the height (µm; nm) and Young’s Modulus (kPa) force maps of analyzed cell regions are displayed. Baseline and Sterile water force maps are taken from the corresponding position (we aimed to record the same region but typically encountered a slight shift during medium exchange) (Supplemental Figure S8).


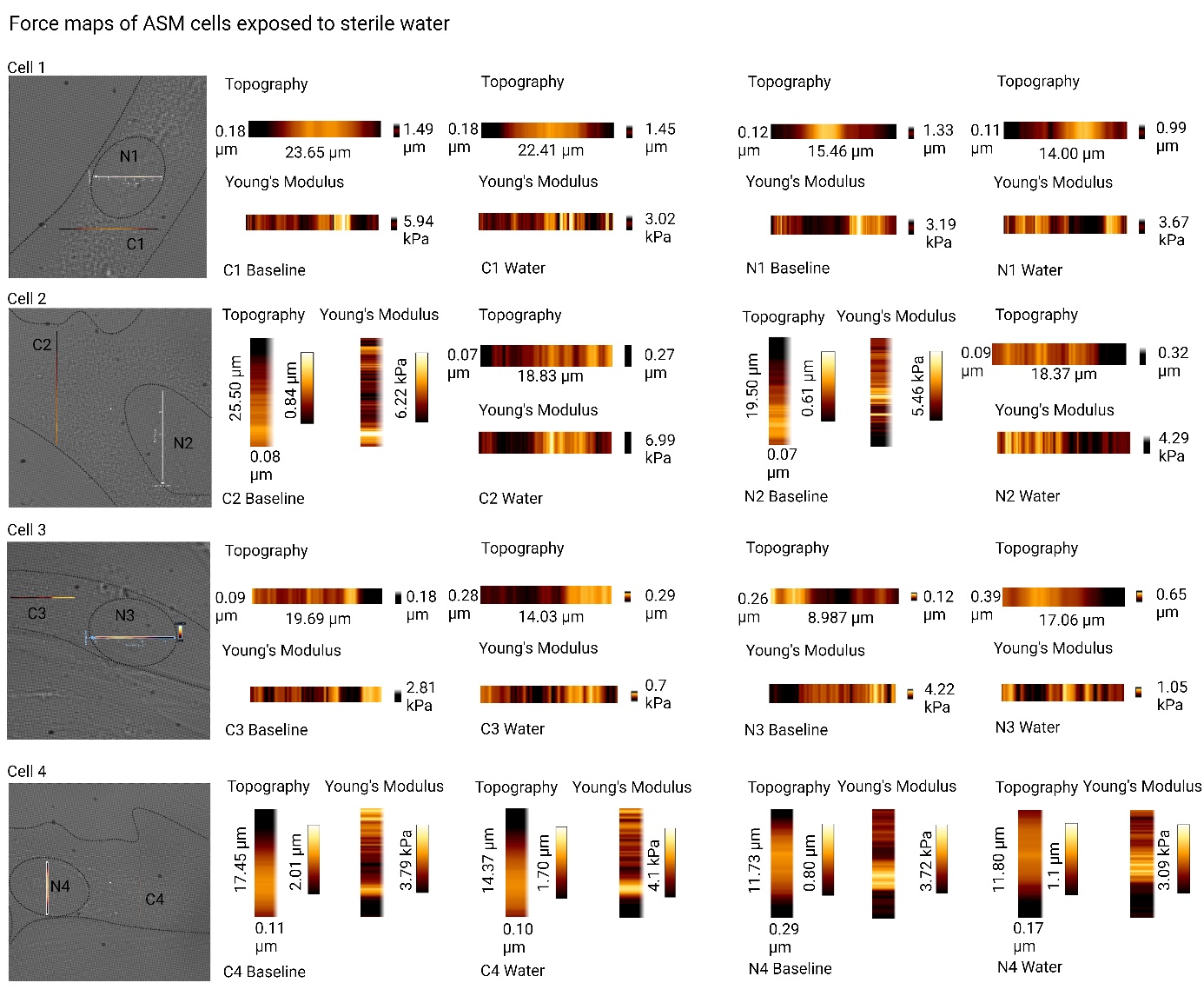


**Supplemental Figure S8.** Force maps of airway smooth muscle cells exposed to sterile water (kPa) collected by atomic force microscope. Light microscope images of the cells (63x objective) are displayed in grey with nucleus and cytoplasm denoting lines. Analyzed cell regions are displayed in red-gold force maps representing the height and Young’s Modulus of the cell regions before (baseline) and after sterile water. Different regions were measured at baseline (vertical) and after sterile water (horizontal) at Cell 2.
