# Supplementary figures and images for: Pulmonary surfactant and prostaglandin E2 in airway smooth muscle relaxation of human and male guinea pigs
Source: Physiol Rep. 2024 Sep 8;12(17):e70026. doi: 10.14814/phy2.70026 (PMC11381196; doi:10.14814/phy2.70026)

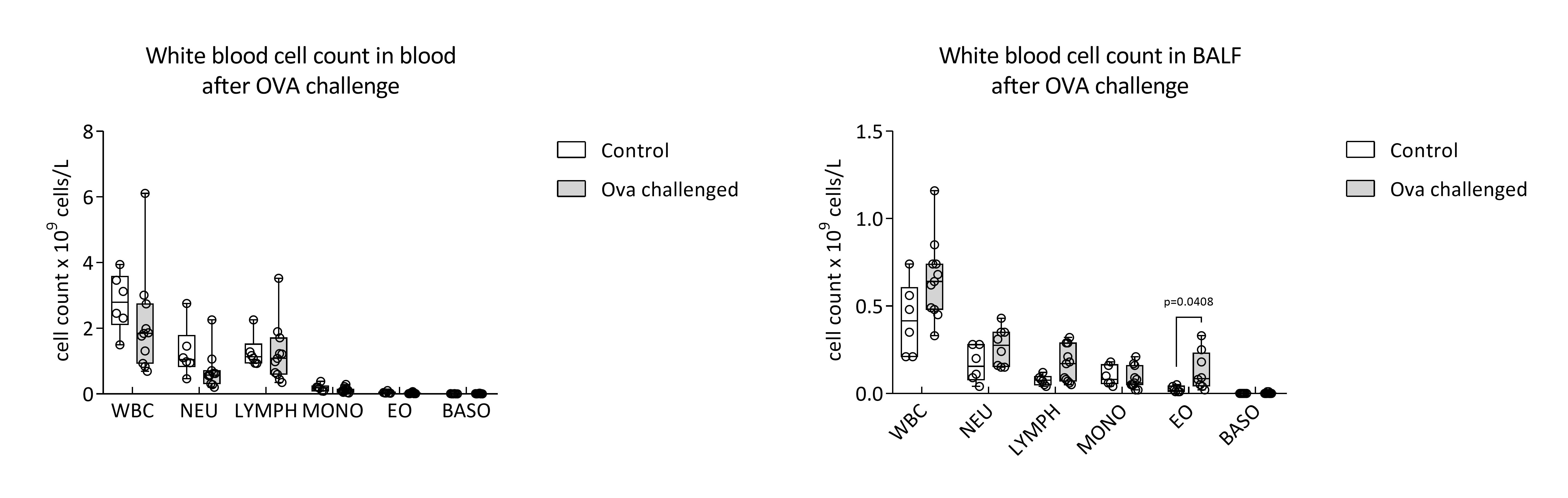

Supplement: Supplementary file 2 — Figure S1. [file PHY2-12-e70026-s006.jpg]

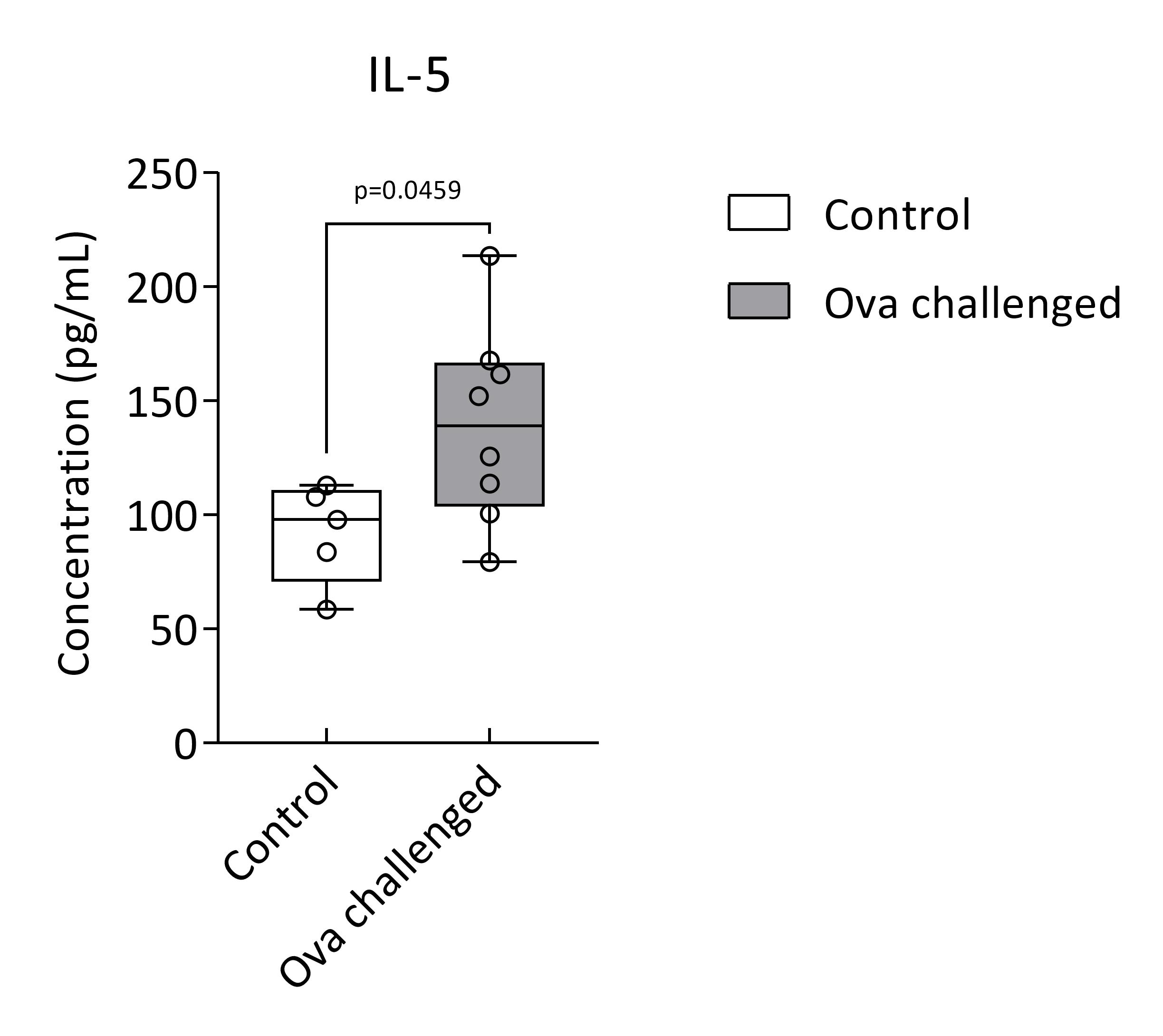

Supplement: Supplementary file 3 — Figure S2. [file PHY2-12-e70026-s003.jpg]

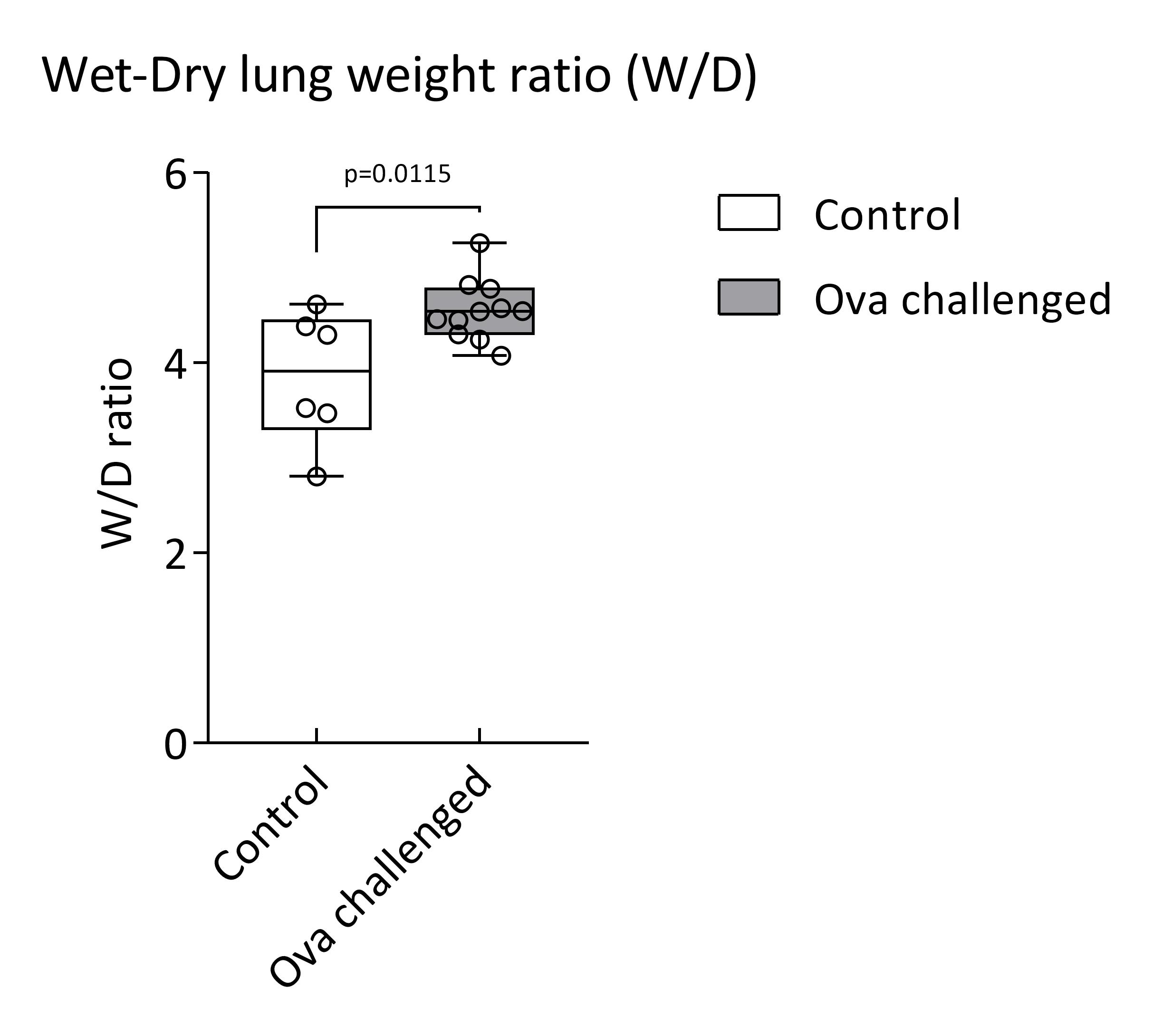

Supplement: Supplementary file 4 — Figure S3. [file PHY2-12-e70026-s007.jpg]

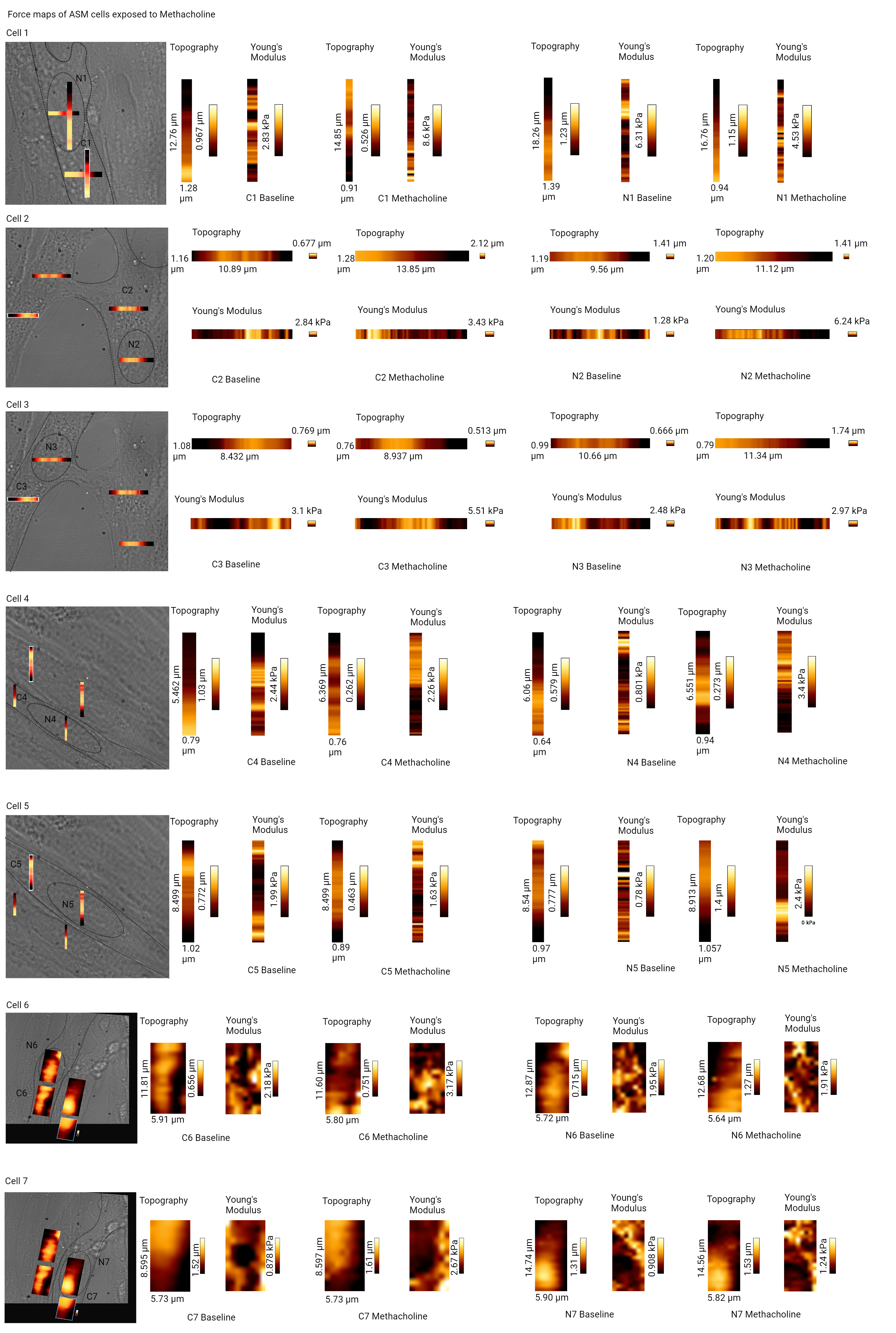

Supplement: Supplementary file 5 — Figure S4. [file PHY2-12-e70026-s009.png]

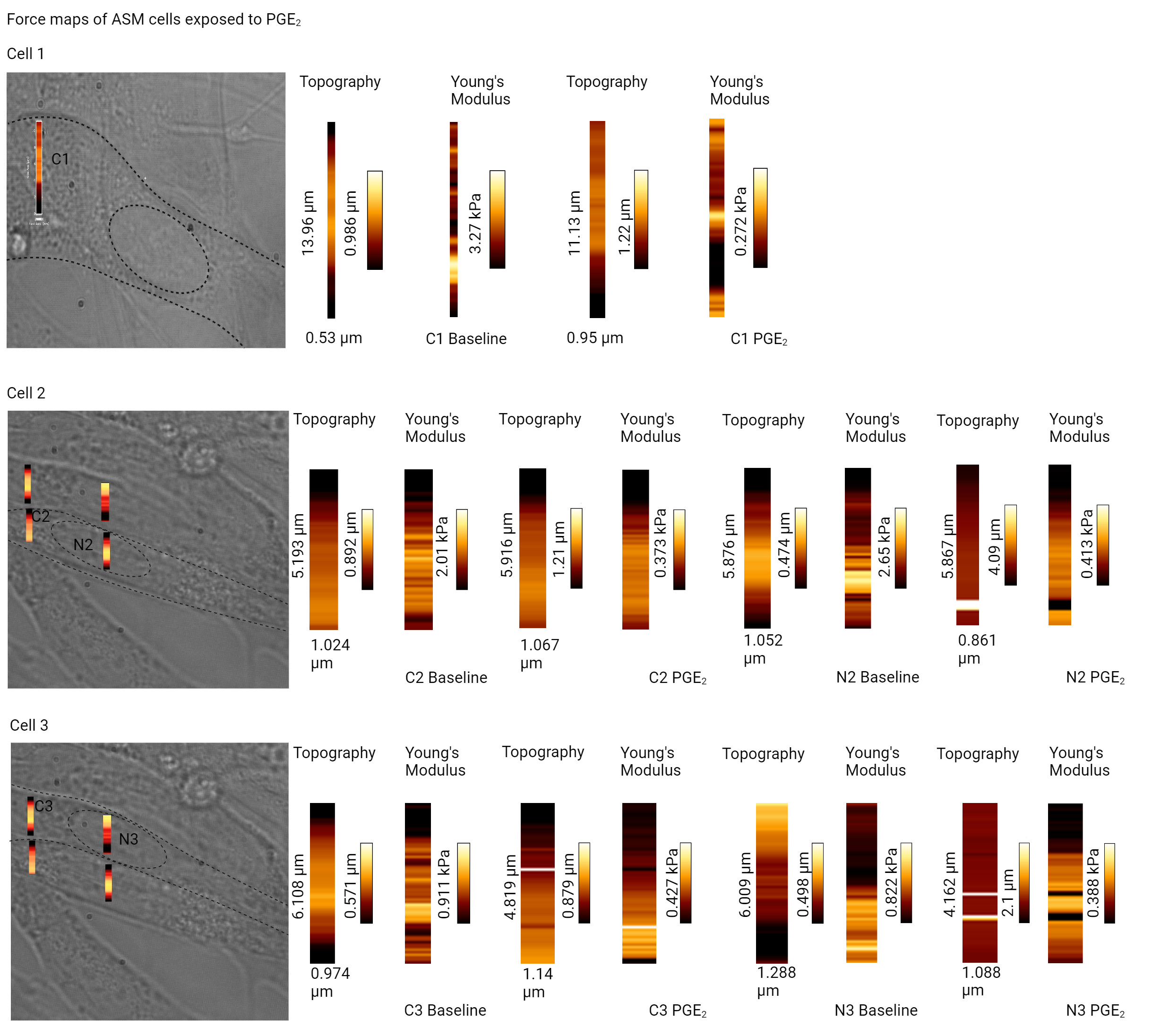

Supplement: Supplementary file 6 — Figure S5. [file PHY2-12-e70026-s002.png]

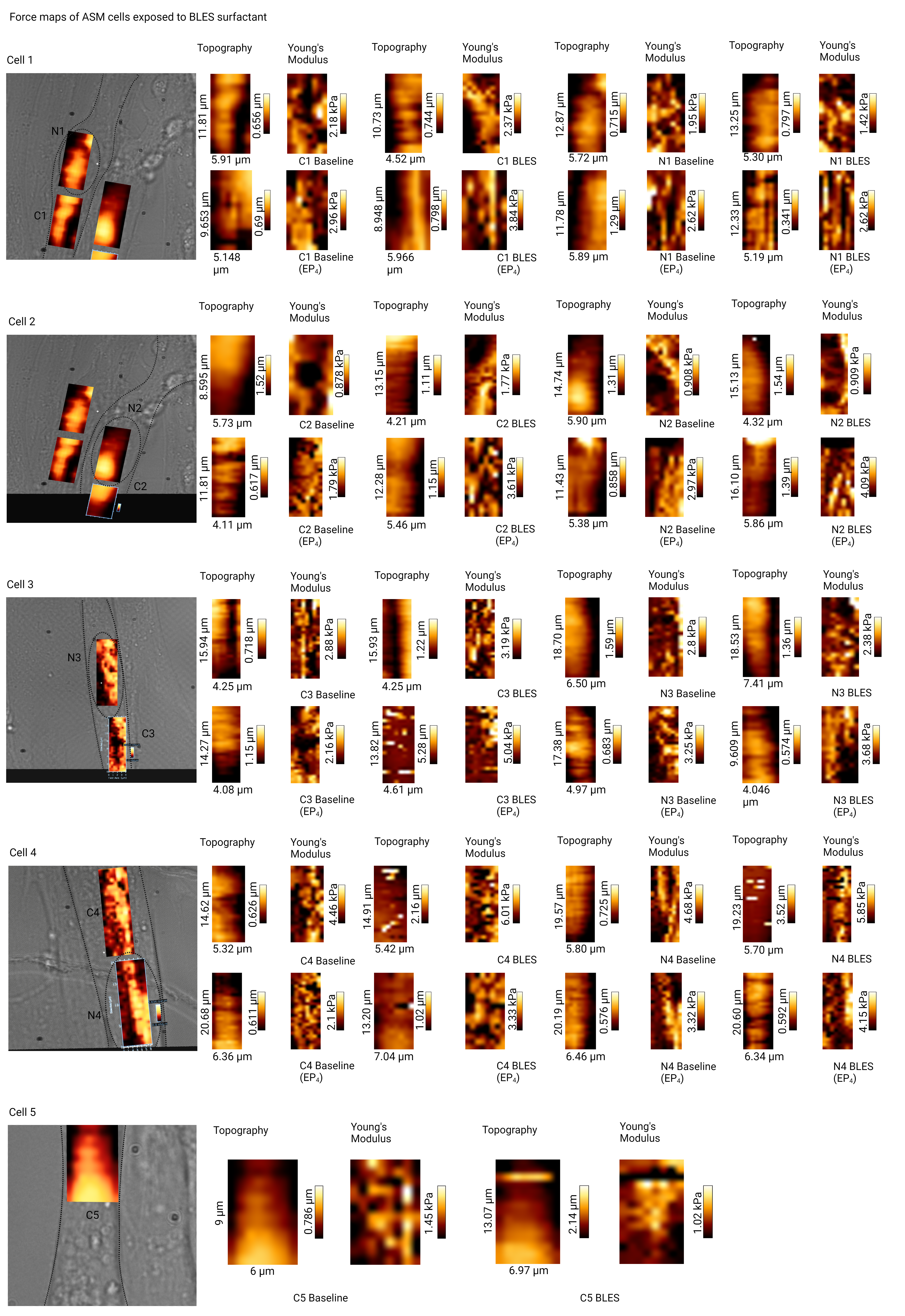

Supplement: Supplementary file 7 — Figure S6. [file PHY2-12-e70026-s004.png]

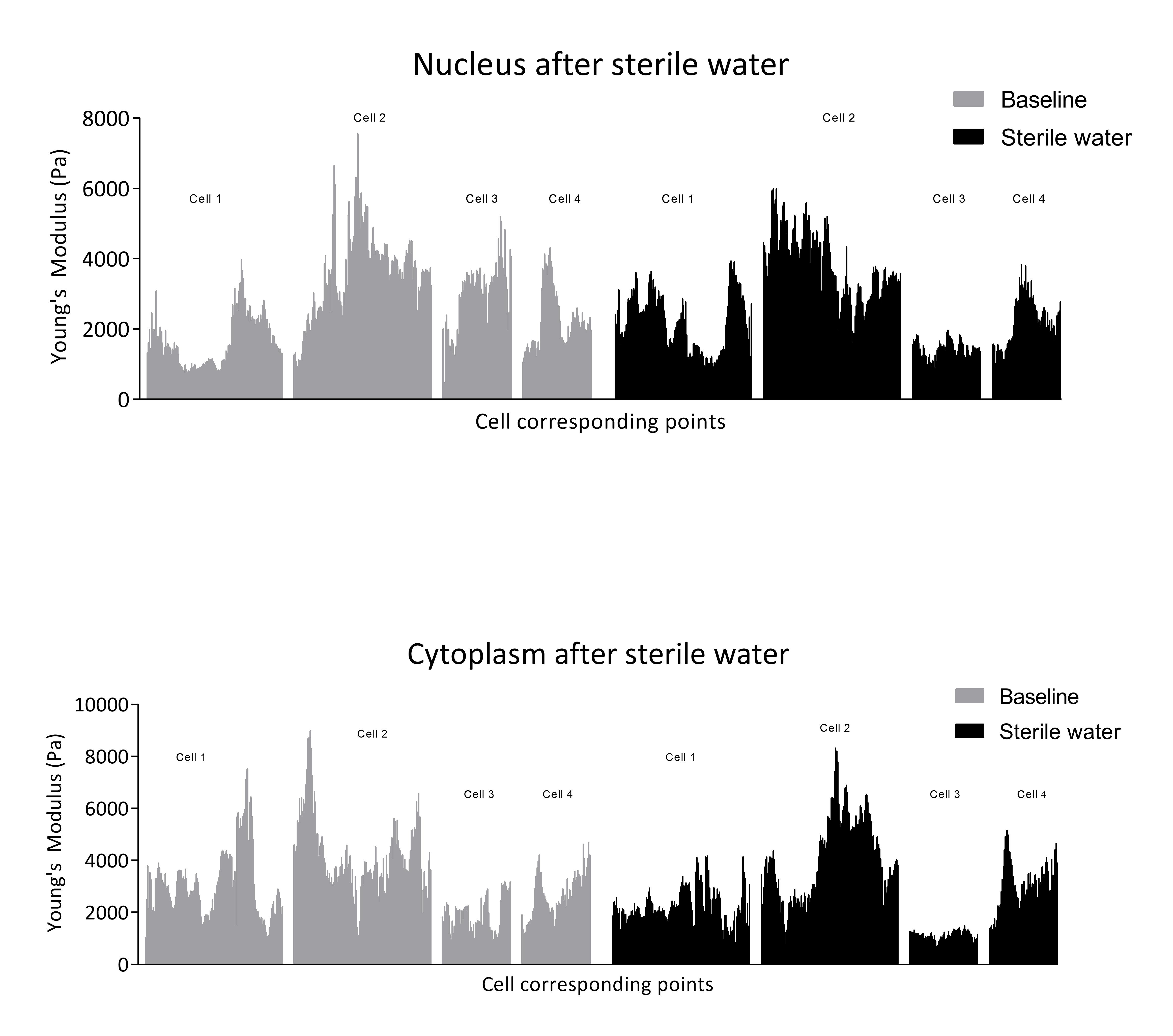

Supplement: Supplementary file 8 — Figure S7. [file PHY2-12-e70026-s005.jpg]

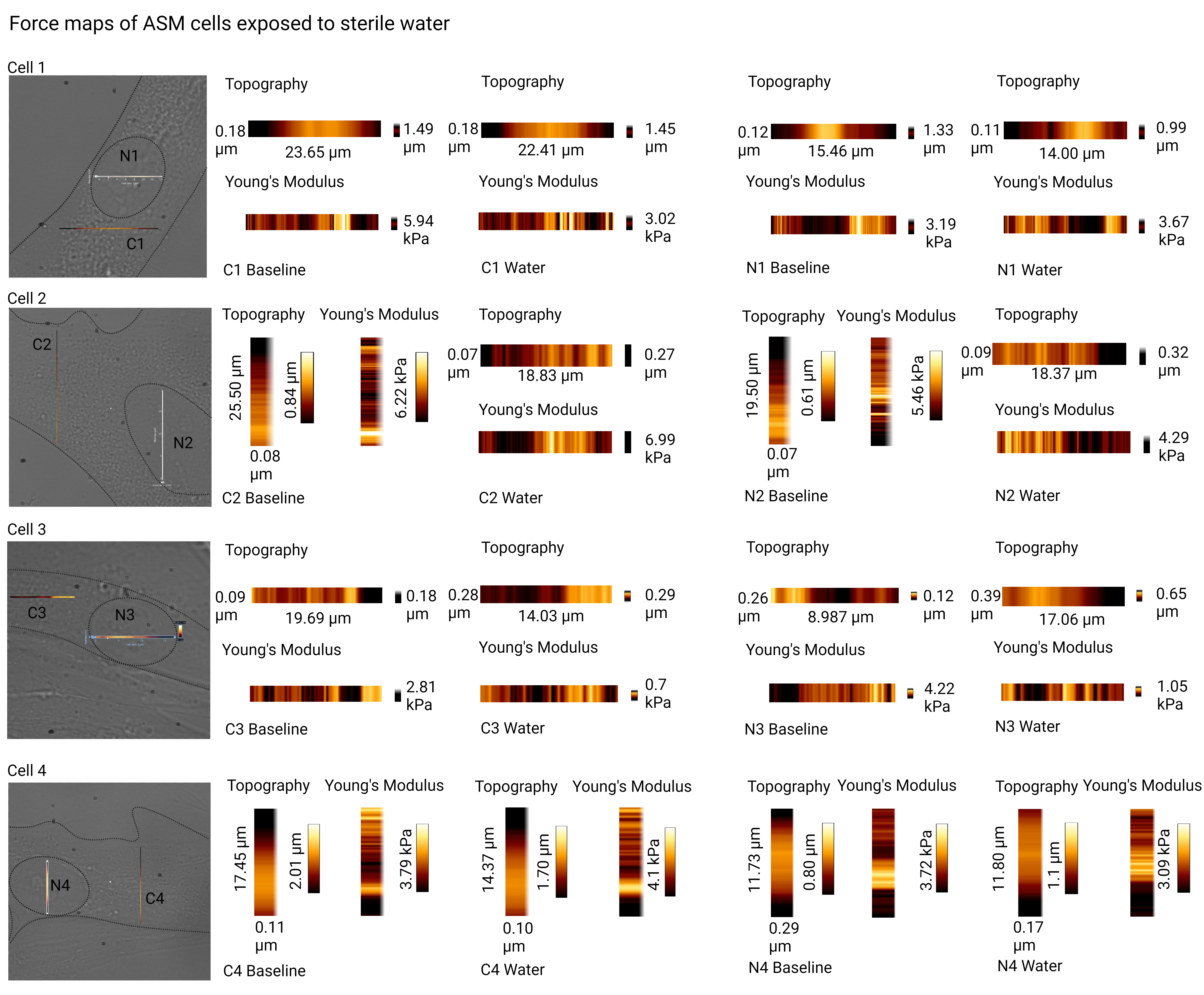

Supplement: Supplementary file 9 — Figure S8. [file PHY2-12-e70026-s008.jpg]
